# Changes in supramolecular organization of cyanobacterial thylakoid membrane complexes in response to far-red light photoacclimation

**DOI:** 10.1126/sciadv.abj4437

**Published:** 2022-02-09

**Authors:** Craig MacGregor-Chatwin, Dennis J. Nürnberg, Philip J. Jackson, Cvetelin Vasilev, Andrew Hitchcock, Ming-Yang Ho, Gaozhong Shen, Christopher J. Gisriel, William H.J. Wood, Moontaha Mahbub, Vera M. Selinger, Matthew P. Johnson, Mark J. Dickman, Alfred William Rutherford, Donald A. Bryant, C. Neil Hunter

**Affiliations:** 1 School of Biosciences, University of Sheffield, Sheffield, UK.; 2 Department of Life Sciences, Imperial College London, London, UK.; 3 Physics Department, Freie Universität Berlin, Berlin, Germany.; 4 Department of Chemical and Biological Engineering, University of Sheffield, Sheffield, UK.; 5 Department of Biochemistry and Molecular Biology, The Pennsylvania State University, University Park, PA, USA.; 6 Department of Life Science, National Taiwan University, Taipei, Taiwan.; 7 Biodesign Center for Applied Structural Discovery, Arizona State University, Tempe, AZ, USA.

## Abstract

Cyanobacteria are ubiquitous in nature and have developed numerous strategies that allow them to live in a diverse range of environments. Certain cyanobacteria synthesize chlorophylls d and f to acclimate to niches enriched in far-red light (FRL) and incorporate paralogous photosynthetic proteins into their photosynthetic apparatus in a process called FRL-induced photoacclimation (FaRLiP). We characterized the macromolecular changes involved in FRL-driven photosynthesis and used atomic force microscopy to examine the supramolecular organization of photosystem I associated with FaRLiP in three cyanobacterial species. Mass spectrometry showed the changes in the proteome of *Chroococcidiopsis thermalis* PCC 7203 that accompany FaRLiP. Fluorescence lifetime imaging microscopy and electron microscopy reveal an altered cellular distribution of photosystem complexes and illustrate the cell-to-cell variability of the FaRLiP response.

## Teaser

Photoacclimation to far-red light changes the oligomeric state of photosystem I in cyanobacterial thylakoid membranes.

## INTRODUCTION

Cyanobacteria are major contributors to primary productivity in marine (*1*) and terrestrial habitats (*2*) and have adapted to occupy almost every environmental niche (*3*). The overwhelming success of these microorganisms rests on the evolution of a diverse range of species, which developed numerous strategies for adapting to a wide range of environmental pressures. One such adaptation is imposed by increased levels of far-red light (FRL), which is found in many environments such as areas shaded by plants, microbial mats, algal blooms, beach rock, tropical soil ecosystems, caves, soil crusts, and stromatolites (*4*–*15*).

Light energy is harvested by three pigment-protein complexes in cyanobacteria: the bilin-containing phycobilisomes and the chlorophyll (Chl)-containing photosystem I (PSI) and PSII complexes (*16*–*18*). It has now been established that some terrestrial cyanobacteria can extend the spectral range for driving oxygenic photosynthesis beyond 700 nm, by synthesizing Chl d and Chl f (*19*–*31*). These pigments confer a competitive advantage in environments enriched in FRL, given that the 600- to 700-nm and 700- to 800-nm wavelength ranges are comparable in terms of the solar output that reaches the surface of Earth (*32*).

There are two distinct cases of adaptation to FRL in cyanobacteria; the first to be characterized was found in the cyanobacterium *Acaryochloris marina*, originally isolated from a biofilm on the undersurface of the didemnid ascidian *Lissoclinum patella* (*19*) and subsequently found in numerous environments enriched in FRL (*33*). *A. marina* uses Chl d as its primary pigment in either white light (WL) or FRL and synthesizes photosystems that contain 90 to 99% Chl d, with Chl a as the minor pigment.

In contrast, some cyanobacteria primarily use Chl a in their photosystems but synthesize Chl d and Chl f as part of a process called FRL-induced photoacclimation (FaRLiP), first characterized in the cyanobacterium *Leptolyngbya* sp. JSC-1 (*7*, *34*) and reviewed in (*9*). In cyanobacteria that perform FaRLiP, the synthesis of Chl d and Chl f in response to FRL is accompanied by extensive remodeling of the photosynthetic apparatus (*7*, *22*, *26*, *29*, *30*); genes encoding paralogs of PSI, PSII, and phycobiliprotein subunits are expressed, and their products are incorporated into pigment-protein complexes.

The gene cluster that mediates FaRLiP is found in many cyanobacteria (*14*, *22*, *35*). It encodes the knotless phytochrome photoreceptor RfpA and response regulators RfpB and RfpC that activate the expression of genes required for remodeling the phycobilisome and photosystems as part of the FaRLiP response (*23*). Within this cluster, the *chlF* gene, which encodes Chl f synthase (*24*, *31*, *36*), is required for the synthesis of the red-shifted Chl f pigment that is incorporated into PSI and PSII (*7*, *23*, *26*, *29*, *30*, *31*, *37*). While the gene(s) encoding the protein complexes required for the final stages of Chl d synthesis have yet to be identified, it has been shown in certain cyanobacteria such as *Chlorogloeopsis fritschii* PCC 9212 (hereafter *C. fritschii* 9212) that increased levels of Chl d synthesis are linked to FaRLiP (*7*, *28*, *38*, *39*). It has also been shown that Chl d is absent from PSI and a single Chl d per PSII is present in *Chroococcidiopsis thermalis* PCC 7203 (hereafter *C. thermalis* 7203) (*26*).

The ultimate functional unit that responds to FRL, or any region of the solar spectrum, is the photosynthetic membrane that contains the two photosystems, as well as the cytochrome *b*_6_*f* complex and other complexes involved in the conversion of solar energy into adenosine 5′-triphosphate (ATP) and reduced nicotinamide adenine dinucleotide phosphate (NADPH), such as the ATP synthase and NADH dehydrogenase-1 (NDH-1) (*3*). The organization of cyanobacterial photosystems and phycobilisomes has been revealed using atomic force microscopy (AFM) and cryo–electron tomography (*40*–*44*), and large changes in the organization of PSI and PSII complexes have been observed in the thylakoid membranes of cyanobacteria undergoing chromatic acclimation (*45*). However, the native organization of the photosystems in FaRLiP cyanobacteria grown under WL, as well as changes in the supramolecular assembly of photosystems associated with FaRLiP, requires further characterization. Here, we use AFM to reveal large-scale alterations in the distribution of PSI induced by FaRLiP in three cyanobacteria: *C. thermalis* 7203, *Synechococcus* sp. PCC 7335 (hereafter *Synechococcus* 7335), and *C. fritschii* 9212. Mass spectrometry was used to identify the changes in the proteome that relate to FaRLiP in *C. thermalis* 7203, particularly those associated with switching PSI from a dimeric configuration in WL to trimers in FRL. Simultaneous fluorescence lifetime imaging microscopy (FLIM) and spectral imaging of WL-grown and FRL-acclimated *C. thermalis* 7203 cells show that FaRLiP affects the distribution of Chl pigments in whole cells.

## RESULTS

### Supramolecular organization of FRL-acclimated and WL-grown thylakoids from *C. thermalis* 7203

**Fig. 1.**
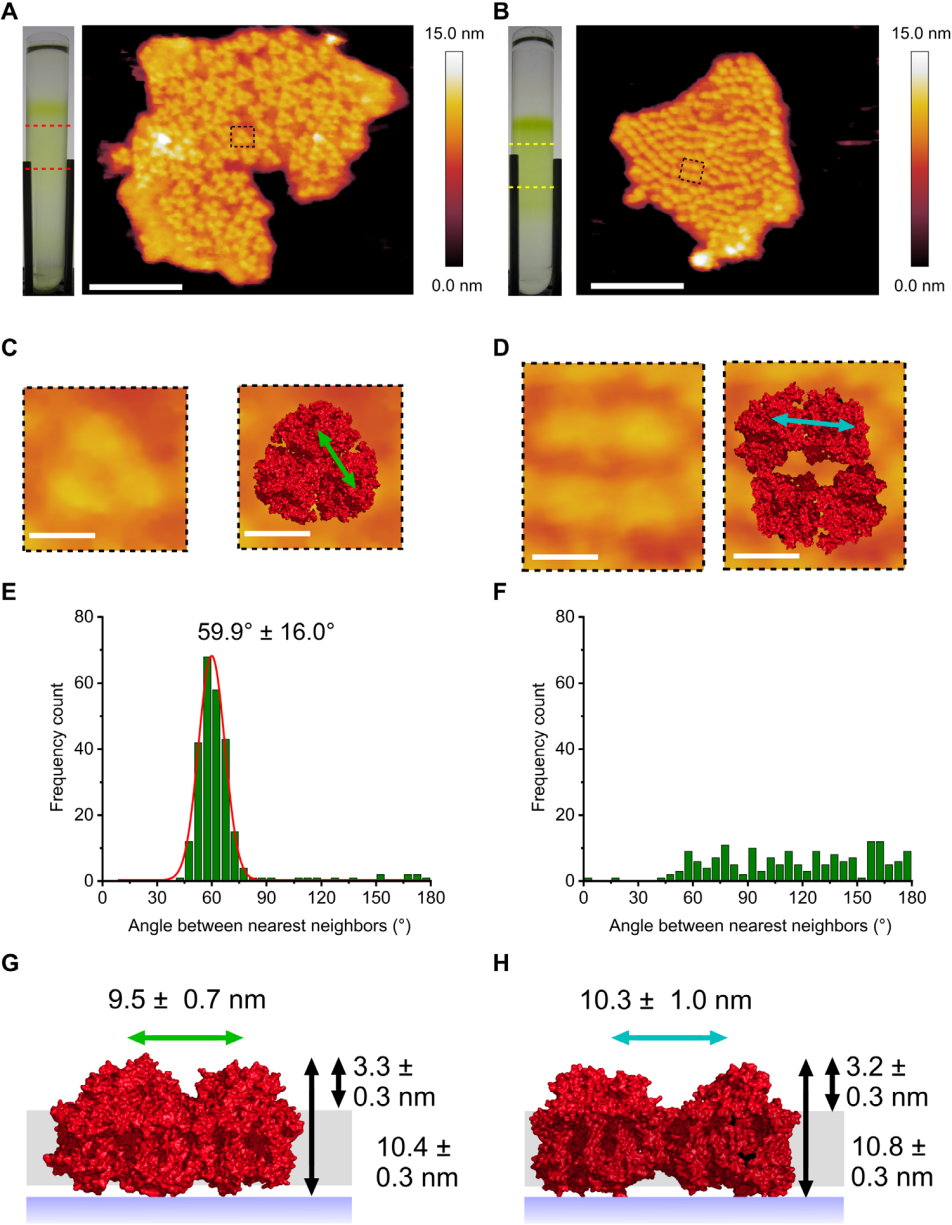
AFM of PSI in thylakoid membranes from FRL-acclimated and WL-grown *C. thermalis* 7203. (**A**) Thylakoid membranes from FRL-acclimated cells purified on sucrose gradients (left) and imaged by AFM (right) revealing trimeric PSI complexes; the red dashed lines delineate the region of the sucrose gradient that samples were harvested from for AFM imaging. (**B**) Thylakoid membranes from cells grown under WL conditions purified on sucrose gradients (left) and imaged by AFM (right) showing densely packed non-trimeric PSI complexes; the yellow dashed lines delineate the region of the sucrose gradient from which thylakoid membranes were harvested for AFM analysis. (**C**) Zoomed-in view of the area outlined in (A) showing the AFM topography of a single trimeric PSI complex (left) and the trimeric cryo–electron microscopy (cryo-EM) structure [Protein Data Bank (PDB) ID: 6PNJ] fitted to the AFM topology (right). (**D**) Zoomed-in view of the area highlighted by the black box in (B) showing the AFM topology of non-trimeric PSI complexes (left) with the PSI cryo-EM structure (PDB ID: 6JEO) fitted to the AFM topograph (right). (**E** and **F**) Histograms showing the angle between the two PSI complexes that are the nearest neighbors for a given PSI complex in (A) and (B), respectively. (**G**) Schematic showing the heights of complexes above the mica surface and the lipid bilayer for FRL membranes and the distance between constituent PSI complexes that make up the trimeric complex (green arrow) and height of PSI complexes above the mica surface (long black arrow) and the lipid bilayer (short black arrow). (**H**) Schematic showing the same distances as in (G) for PSI complexes in membranes from WL-grown cells with the intracomplex distance also shown (blue arrow). Black arrows as in (G). Scale bars, 100 nm (A and B) and 10 nm (C and D).

Thylakoid membranes isolated from *C. thermalis* 7203 cells grown under FRL and WL were fractionated on sucrose gradients and imaged by AFM (Fig. 1). Membranes from cells acclimated to FRL contained arrays of apparently trimeric complexes (Fig. 1A), similar in appearance to AFM topographs of trimeric PSI complexes in thylakoid membranes from several cyanobacteria (*40*, *42*). The trimeric complexes in FRL-acclimated cells had average heights above the mica surface and the lipid bilayer of 10.4 ± 0.3 nm and 3.3 ± 0.3 nm, respectively (Fig. 1G); these dimensions are consistent with the structure of trimeric FRL-PSI from *Fischerella thermalis* PCC 7521 (hereafter *F. thermalis* 7521) and *Halomicronema hongdechloris* (*29*, *30*). A Gaussian distribution fitted to the nearest neighbor analysis had a mean of 59.9° and an SD of 16.0° (Fig. 1E), suggesting that most PSI complexes were in a trimeric configuration (Fig. 1C). The average lateral distance between the constituent monomers that make up the trimeric complexes was 9.5 ± 0.7 nm (Fig. 1, C and G), again consistent with the trimeric PSI structure, so these *C. thermalis* 7203 FRL complexes were assigned as trimeric PSI (Fig. 1C).

No such complexes were imaged in thylakoid membranes from *C. thermalis* 7203 cells grown under WL (Fig. 1B). The average height measurements of these complexes above the mica surface and lipid bilayer were 10.8 ± 0.3 nm and 3.2 ± 0.3 nm, respectively (Fig. 1H), again consistent with the PSI structure. The angle between the two nearest PSI complexes of each individual PSI complex was measured, yielding a seemingly random value (Fig. 1F) rather than the 60° (trimeric) or bimodal 65° and 115° distributions for tetramers (*46*, *47*). The absence of a fixed angle between nearest neighbors is incompatible with a homogeneous population of PSI tetramers. Instead, it suggests that a substantial proportion of the PSI complexes in WL membranes of *C. thermalis* 7203 are in a monomeric or dimeric configuration with a disorganized distribution in the thylakoid membrane. The average lateral distance between PSI complexes is 10.3 ± 1.0 nm, consistent with the structure of one of the dimers within a tetrameric PSI complex (*46*–*51*), and these complexes are therefore assigned as dimers (Fig. 1, D and H). Solubilized thylakoid membranes from WL-grown and FRL-acclimated cells were fractionated on sucrose gradients (fig. S1A), and a prominent green band in the FRL-acclimated sample contained PSI trimers (fig. S1C), consistent with the AFM analysis. Less intense bands containing PSI and PSII monomers and dimers were also observed. The WL sample contained a single green band that appeared to contain monomers or dimers of both PSI and PSII (fig. S1B), again consistent with the AFM images of WL thylakoids. Solubilized thylakoid membranes were also analyzed by blue native polyacrylamide gel electrophoresis (BN-PAGE), which indicated a similar pattern with predominantly PSI dimers and monomers present in the WL sample and PSI trimers present in the FRL-acclimated sample (fig. S1F). This analysis suggests that the AFM topographs are representative of the thylakoid membrane system with PSI trimers, the predominant configuration under FRL conditions and absent under WL conditions. Thus, FaRLiP remodels PSI complexes in *C. thermalis* 7203 and therefore the thylakoid in which they are embedded. However, the packing density is not affected, and the FRL and WL membrane patches in Fig. 1 (A and B) contain 5450 and 5466 PSI monomer equivalents μm^−2^, respectively. Using the number of Chl a and Chl f pigments reported to be bound to the PSI structure from FRL-acclimated *F. thermalis* 7521 and *H. hongdechloris*, which are 83 and 7, respectively (*29*, *30*), and extrapolating to *C. thermalis* 7203, would give Chl a and Chl f densities of 452,350 and 38,150 pigments μm^−2^, respectively. The structure of PSI in *Thermosynechococcus elongatus* grown under WL binds 96 Chl a pigments (*52*, *53*), and using this number would give a density of 524,736 pigments μm^−2^ in *C. thermalis* 7203 cells grown under WL.

### Supramolecular organization of FRL-acclimated and WL-grown thylakoids from *C. fritschii* 9212

**Fig. 2.**
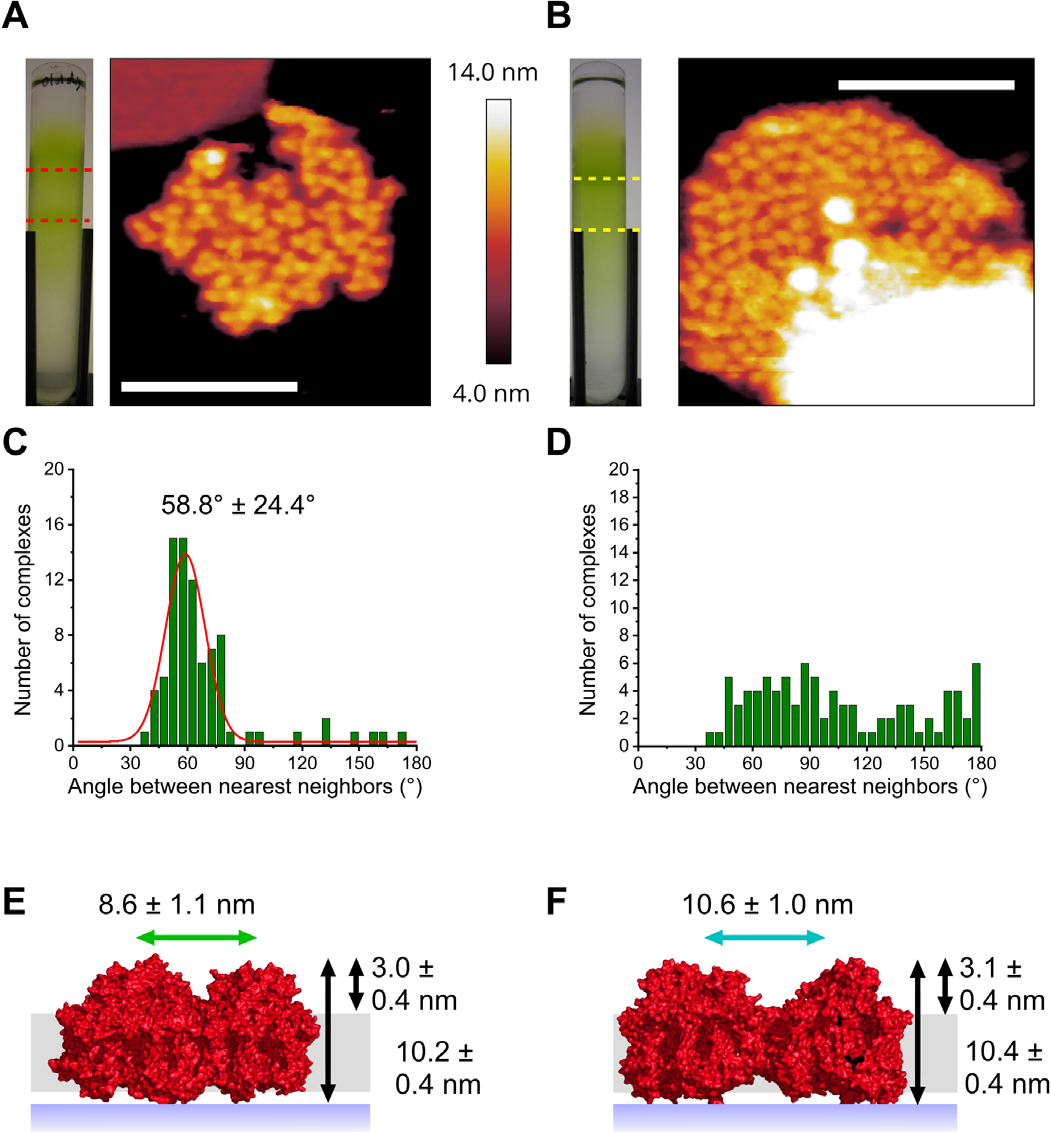
AFM of PSI in thylakoid membranes from FRL-acclimated and WL-grown *C. fritschii* 9212. (**A**) Sucrose gradient of thylakoid membranes purified from FRL-acclimated cells (left) and an AFM topograph of thylakoid membranes showing trimeric PSI complexes (right) harvested from the region indicated by the red dashed lines. (**B**) Sucrose gradient of thylakoid membranes purified from cells grown under WL conditions (left) and an AFM topograph of a thylakoid membrane patch with densely packed PSI complexes visible (right); the yellow dashed lines show where the thylakoid membranes were harvested for AFM imaging. (**C** and **D**) Angles between the two nearest neighbors of each PSI complex in the membrane patches in (A) and (B), respectively. (**E**) Schematic showing the height above the mica surface (long black arrow) and lipid bilayer (short black arrow) measured for PSI complexes in FRL thylakoid membranes (PDB ID: 6PNJ). The internal distance between members of trimeric PSI complexes is also shown (green arrow). (**F**) Schematic of the PSI complexes in WL thylakoid membranes in (B) showing the height above the mica surface (long black arrow) and the lipid bilayer (short black arrow) of PSI complexes (PDB ID: 6JEO). The average distance between neighboring PSI complexes is also shown (blue arrow). Scale bars, 100 nm (A and B).

Because exposure to FRL changes the organization of PSI complexes in thylakoid membranes of *C. thermalis* 7203, another cyanobacterium, *C. fritschii* 9212, was examined with AFM to see whether it responds similarly to FRL. Consistent with *C. thermalis* 7203, trimeric complexes were observed in membranes from the FRL-acclimated cells (Fig. 2A). The average heights above the mica surface and membrane bilayer were 10.2 ± 0.4 nm and 3.0 ± 0.4 nm, respectively (Fig. 2E), consistent with the measurements of the PSI complex (*29*, *30*, *40*). The angle between each complex and the two closest adjacent complexes was measured, and a Gaussian distribution was fitted to the measured angles with an average of 58.8° and an SD of 24.4° (Fig. 2C), indicating that most PSI complexes are trimeric. The average internal distance between members of the trimeric complexes was 8.6 ± 1.1 nm (Fig. 2E), consistent with the trimeric PSI structure and previous AFM data of PSI complexes (*29*, *30*, *40*); thus, these complexes were assigned as trimeric PSI.

The complexes in thylakoid membranes isolated from cells grown in WL were similar in appearance to those from WL-grown *C. thermalis* 7203 (Fig. 2B), with average heights above the mica and membrane bilayer of 10.4 ± 0.4 nm and 3.1 ± 0.4 nm, respectively, and a random distribution of angles between nearest PSI complexes (Fig. 2D). The average distance between adjacent topological features was 10.6 ± 1.0 nm (Fig. 2F), consistent with dimeric PSI (*46*–*51*). Sucrose gradients of solubilized thylakoid membranes from WL- grown and FRL-acclimated cells (fig. S2A) revealed a green band in the FRL-acclimated samples that contained trimeric PSI (fig. S2C). This band was not observed for the WL sample; instead, a faster migrating, lower green band was observed in the WL sucrose gradient. This minor fraction was negatively stained and imaged via transmission electron microscopy (TEM) revealing tetrameric PSI complexes together with some dissociated monomeric and dimeric PSI (fig. S2B). These observations suggest that the AFM data are representative of the thylakoid membrane system, with trimeric PSI predominating in FRL-acclimated samples. However, thylakoids from cells grown in WL harbor a substantial proportion of PSI complexes in a tetrameric configuration, in addition to dimeric and monomeric PSI complexes. As observed for FaRLiP in *C. thermalis* 7203 cells, PSI becomes trimeric under FRL, but the packing densities of 5833 (FRL) and 5663 (WL) PSI monomer equivalents μm^−2^ show that there is no appreciable effect of this reorganization on the density of PSI complexes in the thylakoid membrane. Using the number of pigments reported to bind to PSI from FRL-acclimated *F. thermalis* 7521 and *H. hongdechloris* would give Chl a and Chl f densities of 484,139 and 40,831 pigments μm^−2^, respectively, for FRL-acclimated *C. fritschii* 9212 membranes. Using the number of pigments that bind to PSI from *T. elongatus* grown under WL, as well as extrapolating to *C. fritschii* 9212, gives a Chl a density of 542,648 pigments μm^−2^ for membranes from WL-grown cells.

### Supramolecular organization of FRL-acclimated and WL-grown thylakoids from *Synechococcus* 7335

**Fig. 3.**
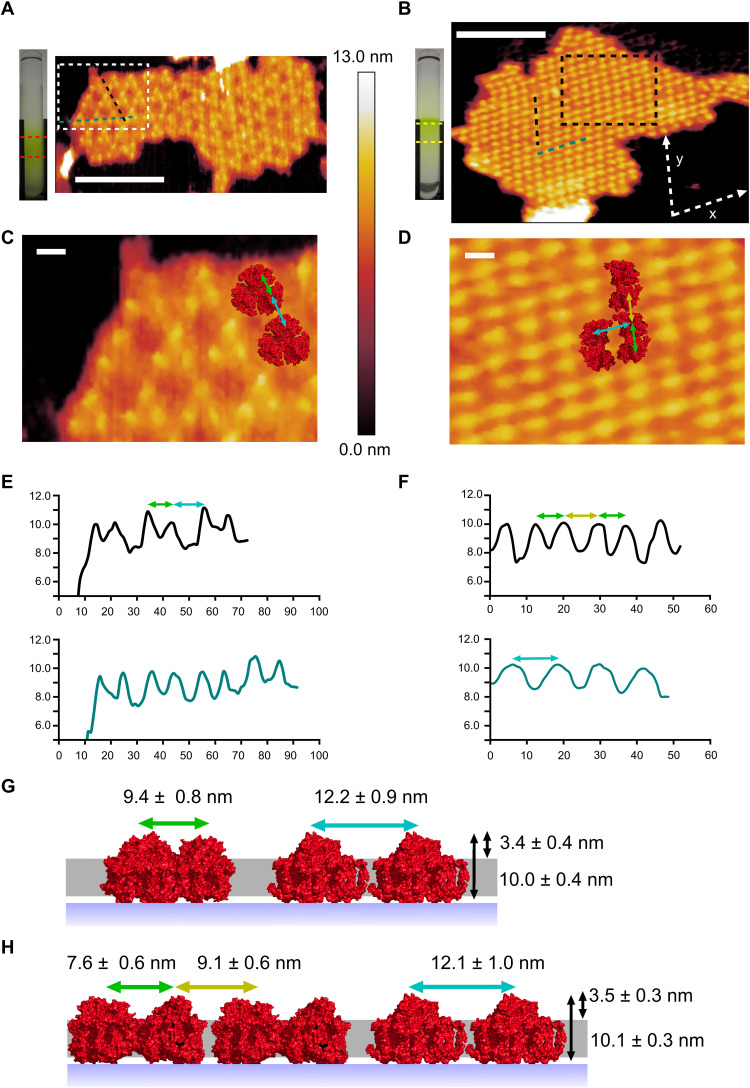
AFM of PSI in thylakoid membranes from FRL-acclimated and WL-grown *Synechococcus* 7335 cells. (**A**) Thylakoid membranes from FRL-acclimated cells purified on a sucrose gradient (left) and imaged by AFM (right) showing a pseudo-hexagonal lattice of PSI trimers; the red dashed lines represent the areas that membranes were selected from for AFM imaging. The black and dark blue dashed lines correspond to the height sections in (E). Scale bar, 100 nm. (**B**) Membranes purified from WL-grown cells were fractioned on a sucrose gradient (left) and imaged by AFM (right); the yellow dotted lines show the location the membranes were isolated from in the sucrose gradient. The dashed lines correspond to the height sections in (F). Scale bar, 100 nm. (**C**) Topograph of the area indicated by the white box in (A) showing trimeric FRL-acclimated PSI (PDB ID: 6PNJ) fitted to the AFM data. The colored arrows correspond to the distances in (E) and (G). Scale bar, 10 nm. (**D**) Topograph of the area delineated by the black box in (B); a dimer unit within a PSI tetramer (PDB ID: 6JEO) was fitted to the AFM data. The colored arrows correspond to those in (F) and (H). Scale bar, 10 nm. (**E** and **F**) Height sections for FRL PSI and WL PSI complexes in (A) and (B), respectively. (**G**) Diagram of the average height of FRL PSI complexes in (A, C) above the mica surface (long black arrow) and the lipid bilayer (short black arrow), the average distance between constituent members of the trimeric PSI complex (green arrow), and the periodicity of the pseudo-hexagonal lattice (blue arrow). (**H**) Diagram for WL PSI complexes in (B, D), black arrows as in (G); PSI intradimer (green arrow) and interdimer (yellow and blue arrows) distances are indicated.

AFM was used to image thylakoid membranes from a third cyanobacterium capable of FaRLiP, *Synechococcus* 7335, which had been grown under FRL and WL. Thylakoid membranes were fractionated on sucrose gradients and imaged by AFM (Fig. 3). FRL-acclimated membranes were composed of a pseudo-hexagonal lattice of trimeric complexes (Fig. 3A), similar in appearance to AFM topographs of trimeric PSI complexes in thylakoid membranes from several cyanobacteria (*40*, *42*) and to those described above. The average heights above the mica surface and the lipid bilayer of these complexes, 10.0 ± 0.4 nm and 3.4 ± 0.4 nm (Fig. 3G), respectively, are consistent with the crystal and cryo–electron microscopy (cryo-EM) structures for PSI (*17*, *29*, *30*, *37*, *52*, *53*) and with previous AFM measurements of PSI in lipid bilayers (*40*–*42*, *44*, *45*). The average lateral distance between the constituent PSI monomers that make up the trimeric PSI complex was 9.4 ± 0.8 nm (Fig. 3, C, E, and G), again consistent with previous measurements. The periodicity of this pseudo-hexagonal lattice was 12.2 ± 0.9 nm (Fig. 3, C, E, and G), comparable to the organization of PSI trimers previously observed in cyanobacterial thylakoid membranes (*40*, *42*, *45*). Therefore, these complexes were assigned as trimeric PSI complexes (Fig. 3C).

AFM reveals a different organization of PSI complexes in membranes isolated from WL-grown cells (Fig. 3B), similar to that observed for PSI complexes in membranes from red light (RL)–grown cells (*45*). The average heights of protein complexes above the mica surface and lipid bilayer were 10.1 ± 0.3 nm and 3.5 ± 0.3 nm (Fig. 3H), respectively, almost identical to the heights measured for the PSI complexes in the FRL-acclimated membrane patches. There are three distinct spacings between individual topological features in the lattice of 12.1 ± 1.0 nm in the *x* direction defined in Fig. 3B (Fig. 3, D, F, and H), and with alternating “long” (9.1 ± 0.6 nm) and “short” spacings (7.6 ± 0.6 nm) in the *y* direction (Fig. 3, D, F, and H). These lateral distances are consistent with either dimeric or tetrameric PSI purified from *Chroococcidiopsis* sp. TS-821 and *Nostoc* sp. PCC 7120 (hereafter *Nostoc* 7120) (*46*–*51*). However, the alternate long and short spacings are likely indicative of a dimeric PSI complex, with the shorter spacing the result of the interaction between the two individual PSI monomers in a dimer. Accordingly, the data are modeled as a lattice of dimeric PSI complexes (Fig. 3D).

While the predominant arrangement of PSI seen in the AFM data from WL-grown cells appeared to be dimeric, it was possible to image some PSI complexes in a trimeric configuration, albeit in a disorganized state and not in a pseudo-crystalline array (fig. S3A). Sucrose gradients of solubilized membranes suggested that monomeric, dimeric, trimeric, and even tetrameric complexes are all present in cells grown in WL (fig. S3B). In sucrose gradients of solubilized thylakoid membranes from FRL-acclimated cells, the most prominent green band corresponds to trimeric PSI, suggesting that the AFM data are representative of the thylakoid membrane system; however, other green bands are present that correspond to dimeric and monomeric complexes. There does appear to be an increase in the proportion of PSI complexes that adopt a trimeric configuration in *Synechococcus* 7335 (fig. S3B), as is the case with *C. thermalis* 7203 and *C. fritschii* 9212. However, the presence of substantial proportions of dimeric and trimeric PSI in FRL-acclimated and WL-grown samples, respectively, suggests that the reorganization of PSI complexes during FaRLiP in *Synechococcus* 7335 is not as extensive as in *C. thermalis* 7203 and *C. fritschii* 9212. The densities of PSI complexes in the FRL and WL membrane patches in Fig. 3 (A and B) are 6614 and 6849 PSI monomer equivalents μm^−2^, respectively. This would result in Chl a and Chl f densities of 548,962 and 46,298 pigments μm^−2^, respectively, in FRL-acclimated membranes and a Chl a density of 657,504 pigments μm^−2^ in WL-grown cells. These data indicate that the change in PSI organization between WL-grown and FRL-acclimated cells largely does not alter the density of PSI complexes in the thylakoid membranes although it does change the densities of Chl a and Chl f pigments in the membrane. The membrane packing density of PSI was ~20% higher in this high-light sensitive strain than observed in *C. thermalis* 7203 and *C. fritschii* 9212. In summary, AFM shows that FaRLiP induces large-scale alterations in the content and membrane organization of PSI in the thylakoids of *C. thermalis* 7203, *C. fritschii* 9212, and *Synechococcus* 7335, revealing a remarkable consistency in response to FRL in these three cyanobacteria.

### Proteomic analysis of WL-grown and FRL-acclimated thylakoids from *C. thermalis* 7203 by mass spectrometry

Thylakoid membrane proteins from WL-grown and FRL-acclimated *C. thermalis* 7203 were analyzed by mass spectrometry to determine the changes in the proteome associated with FaRLiP and to reveal a possible explanation for the large-scale reorganization of protein complexes in the thylakoid membranes observed by AFM. A widely used label-free protein quantification (LFQ) method, first validated as a proxy for molar amounts and referred to as iBAQ (intensity-based absolute quantification) (*54*), was used for a global comparison of the datasets. Intra-WL and -FRL correlation matrices (fig. S4) show Spearman rank correlation coefficients of 0.97 to 0.98 with a marked contrast in the numbers of proteins identified in replicates of WL and FRL thylakoid membrane samples of 272 to 284 and 575 to 580, respectively. Alongside this twofold increase in the number of thylakoid proteins identified after FRL acclimation, 403 of the 629 proteins detected overall had iBAQ abundance scores that were significantly different between the two growth conditions (*P* < 0.05) (table S1).

**Fig. 4.**
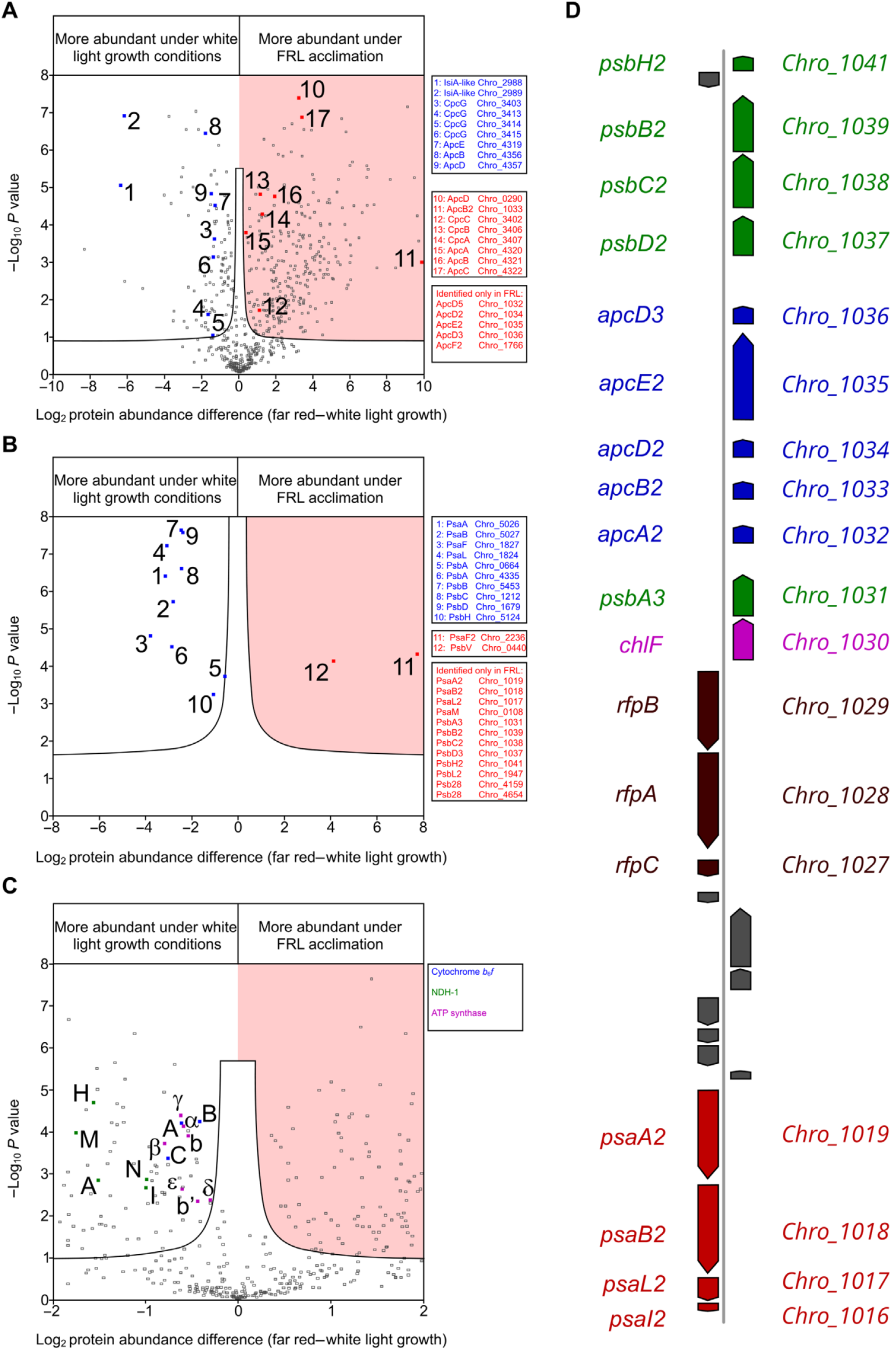
The effect of WL growth and FRL acclimation on the relative distribution of light-harvesting, photosystem, electron transport and ATP synthase subunits from *C. thermalis* 7203. (**A**) Volcano plot showing the WL/FRL distributions of thylakoid proteins quantified by iBAQ. The data points are listed in data S1 together with details of the statistical analysis. The threshold of significance at *P* = 0.05 is indicated by a black line. Light-harvesting antenna proteins predominating under WL growth conditions are shown in blue points and those at relatively higher levels under FRL as red points shaded by a red background. Proteins that were only detected in the FRL group are not shown on the volcano plot but are listed alongside. (**B**) Relative distributions of PSI (Psa) and PSII (Psb) subunits quantified by the Top-N method to account for the occurrence of shared tryptic peptides resulting from shared sequence identity (see fig. S5). The data points are listed in data S2. (**C**) Relative distributions of thylakoid proteins quantified by iBAQ shown on an expanded *x*-axis scale. All highlighted subunits are more abundant in cells grown under WL: cytochrome *b*_6_*f* (PetA, PetB, and PetC in blue), NDH-1 (NdhA, NdhH, NdhI, NdhM, and NdhN in green), and ATP synthase (α, β, γ, δ, ε, b, and b′ in magenta). (**D**) FaRLiP gene cluster from *C. thermalis* 7203 showing PSI subunits (red), PSII subunits (green), phycobiliprotein subunits (blue), regulatory genes (brown), and Chl f synthase (magenta). The gene name is on the left of the gene cluster, and the locus tag is on the right.

Statistical analysis of iBAQ abundance scores by a modified *t* test is shown by the volcano plot in Fig. 4A. The phycobiliprotein subunits highlighted in blue (numbered 3 to 9) were detected at two to four times higher abundance in WL compared to FRL. The two antenna proteins Chro_2988 and Chro_2989 were significantly more abundant in WL by a factor of approximately 80. Chro_2988 is predicted to be a Chl-binding protein that has homology with both the IsiA and PsaL proteins and has been hypothesized to act as a membrane-intrinsic ancillary light-harvesting protein to PSI (*55*, *56*). The interaction between the Chro_2988 protein and the PSI complex is hypothesized to be between the PsaL subunit of PSI and the PsaL-like domain of the Chro_2988 protein, which would prevent the formation of the trimeric PSI complex. Chro_2989 also has homology with the IsiA protein, although it lacks the PsaL-like domain seen in the Chro_2988 protein and it likely acts as an ancillary light-harvesting protein. The change in the abundance of Chro_2988 likely plays a role in the large-scale reorganization of PSI observed in the AFM data; in WL, the high abundance of this protein prevents PSI trimer formation, whereas the much lower abundance in FRL allows the PsaL2 subunits (Chro_1017) of PSI monomers to interact and form trimers.

Highlighted in red in Fig. 4A are phycobiliprotein subunits that were found to predominate in cells acclimated to FRL by a factor of between 2 and 10. At the extreme, Chro_1033 was approximately 1000 times more abundant in FRL, and the other FaRLiP gene cluster–encoded phycobiliprotein subunits were detectable only in FRL (Fig. 4A). The only non-FaRLiP gene cluster phycobiliprotein subunit found exclusively in FRL was an ApcF2 paralog (Chro_1766), which is consistent with previous proteomics studies of phycobilisome subunits under FaRLiP in *C. thermalis* 7203 (*57*).

Quantification by iBAQ is based on the summed intensities of all the tryptic peptide ions that belong to a particular protein. However, many of the photosystem subunit paralogs identified in this study have regions with a high degree of sequence identity and therefore contain shared tryptic peptides. In cases where shared peptide ion intensities are composed of combined signals from paralogs occurring at significantly different expression levels, iBAQ would introduce quantitative inaccuracy. For example, PsaA paralogs Chro_5026 and Chro_1019 and PsaB paralogs Chro_5027 and Chro_1018 have four and six shared tryptic peptides (6 to 30 residues), respectively (for alignments, see fig. S5). To address this potential issue, we used an alternative LFQ approach, also validated as a proxy for molar amount (*58*), which uses the summed ion intensities of the three highest unique tryptic peptides.

PSI polypeptides PsaA1 (Chro_5026), PsaB1 (Chro_5027), PsaL1 (Chro_1824), and PsaF1 (Chro_1827) were detected in both WL and FRL thylakoids but at 7- to 14-fold greater abundance in WL (Fig. 4B). The PSI paralogs encoded in the FaRLiP gene cluster, PsaA2 (Chro_1019), PsaB2 (Chro_1018), and PsaL2 (Chro_1017), were all exclusively identified in FRL (Fig. 4B). While not encoded in the FaRLiP gene cluster, a PsaF2 paralog (Chro_2236) was predominantly present in FRL, and it was only represented in WL by a single, low-intensity peptide. The PsaI2 (Chro_1016) subunit encoded on the FaRLiP gene cluster was not detected in the FRL sample owing to a lack of suitable tryptic peptides; a similar issue prevented the detection of PsaJ2 (Chro_2235).

The same pattern was apparent with PSII subunits, with all of the paralogs of PsbA (D1), PsbD (D2), PsbC1 (CP43), PsbB1 (CP47), and PsbH1 not encoded in the FaRLiP gene cluster detected at 1.5- to 7-fold lower abundance in FRL than WL (Fig. 4B). The FaRLiP gene cluster–encoded paralogs were exclusively identified in the FRL samples. The only FaRLiP gene cluster–encoded protein that was not detected in the FRL membranes was the ChlF protein (Chro_1030), which was not identified in either the WL or FRL sample. This finding was somewhat unexpected as the ChlF protein has been assigned as the Chl f synthase (*24*, *36*). Encoded externally to the FaRLiP gene cluster, a PsbL paralog (Chro_1947) and the two Psb28 paralogs (Chro_4159 and Chro_4654) were also exclusive to FRL, and PsbV (Chro_0440) was detected in both WL and FRL in a 1:18 ratio.

We also analyzed the change in abundance of the ATP synthase and the other membrane bound proteins that make up the photosynthetic electron transport chain (PETC) (using the iBAQ method) and found all of these proteins to be at a higher abundance in the thylakoid membranes from the WL growth conditions relative to FRL-acclimated cells (Fig. 4C). The ATP synthase subunits were found to be between 1.2 and 1.7 times more abundant in WL, and the subunits for the cytochrome *b*_6_*f* and NDH-1 complexes were 1.3 to 1.7 and 2.0 to 3.4 times more abundant in WL, respectively (Fig. 4C).

### Spectral and fluorescence lifetime imaging and electron microscopy of WL-grown and FRL-acclimated *C. thermalis* 7203

**Fig. 5.**
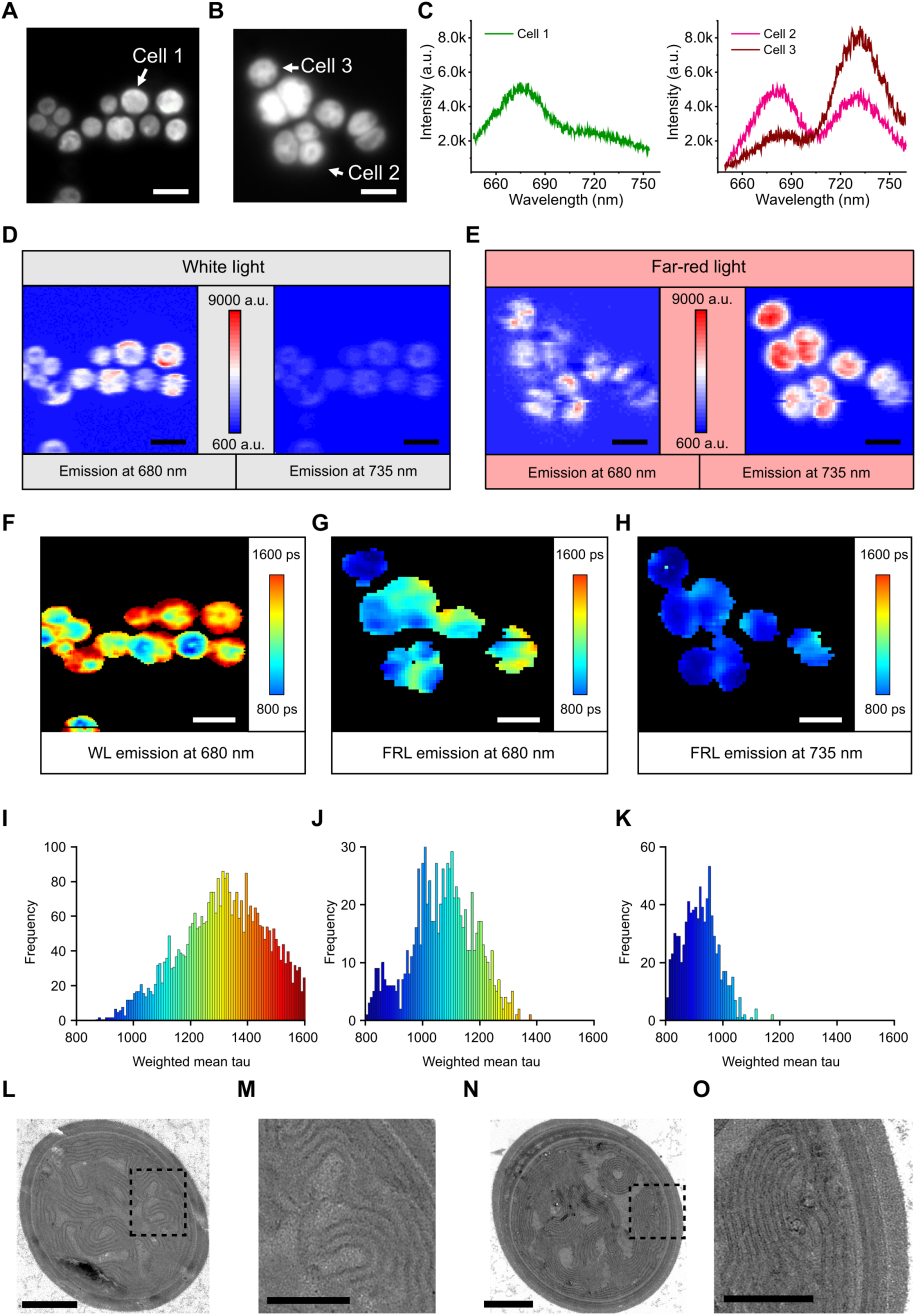
Functional and structural imaging of *C. thermalis* 7203 cells grown under WL and FRL. (**A**) False-color fluorescence images of WL cells. Scale bar, 4 μm. (**B**) False-color fluorescence images of FRL cells. Scale bar, 4 μm. (**C**) Left: Individual emission spectrum recorded from a single WL cell in (A) showing a peak emission at 680 nm. Right: Emission spectra of two FRL cells in (B) showing a peak at 680 nm and an additional peak at 735 nm. (**D**) Spectral maps of WL cells showing the emission intensity at 680 nm (left) and 735 nm (right); the strong signal at 680 nm is due to the maximal emission intensity of Chl a at this wavelength, and the much weaker signal at 735 nm is due to the weak emission of Chl a at this wavelength. Scale bars, 4 μm. (**E**) Spectral maps of FRL cells showing the emission intensity at 680 nm (left) and 735 nm (right); the strong signal at 680 nm is due to the presence of Chl a in FRL cells, and the much stronger signal at 735 nm relative to WL cells is due to the presence of Chl f, which maximally emits at this wavelength. Scale bars, 4 μm. (**F** to **H**) Cellular distribution of amplitude-weighted average lifetime images obtained at 680 nm for WL cells, 680 nm for FRL cells, and 735 nm for FRL cells, respectively. Scale bars, 4 μm. (**I** to **K**) Distribution of the lifetime values from (F) to (H), respectively, with the mean and SD of 1315 ± 121 ps for WL cells at 680 nm, 1090 ± 93 ps for FRL cells at 680 nm, and 964 ± 55 ps for FRL cells at 735 nm. (**L**) Electron micrograph of a thin section from WL cells. Scale bar, 1 μm. (**M**) Zoomed view of the area highlighted by the black square in (L) showing membrane spacings and morphology in more detail. Scale bar, 500 nm. (**N**) Electron micrograph of thin sections from FRL cells. Scale bar, 1 μm. (**O**) Zoomed view of the area highlighted by the black square in (N) showing a reduced spacing between adjacent thylakoid membranes relative to WL cells that is typical of FRL cells. Scale bar, 500 nm.

FaRLiP involves remodeling of PSI complexes to form trimers (Figs. 1 to 3) and large-scale alterations in the proteome to adjust the levels of light-absorbing, reaction center and electron transfer components (Fig. 4). To focus on changes at the level of whole cells and to investigate any heterogeneity in the cellular distribution of Chl a and Chl f, as well as FRL phycobilisomes, live cells of WL-grown and FRL-acclimated *C. thermalis* 7203 were immobilized on agarose gel pads and imaged under near-physiological conditions. The samples were characterized by spectral and fluorescence lifetime imaging in a home-built FLIM instrument optimized for the far-red and near-infrared ranges of the spectrum. False-color fluorescence images of the WL (Fig. 5A) and FRL (Fig. 5B) samples, acquired in epifluorescence mode and illuminated by the 470-nm light-emitting diode (LED) source, show the distribution of the cells on the sample surface. Figure 5C shows the emission spectrum of a single WL cell, with an emission maximum at 680 nm, whereas two FRL cells (Fig. 5C) have an additional emission peak, of variable amplitude relative to 680-nm emission, at 735 nm. When switching to scanning confocal mode and using the 485-nm pulsed laser as the excitation light source, we were able to obtain spectral maps of the cells, in which a complete emission spectrum is recorded for each pixel of the image. Depending on cell size, we collect 300 to 350 data points, in this case emission spectra, per cell, sufficient to map the distribution of emitters, with further specificity conferred by selective excitation and tightly filtered emission of ±6 nm around the central wavelength selected by the monochromator. The spatial distributions of the emission intensities for cells grown under WL and FRL are shown in Fig. 5 (D and E, respectively), for emission intensities at 680 nm (left) and 735 nm (right), corresponding to Chl a and Chl f, respectively. Spectral imaging in Fig. 5 (D and E) reflects the emission spectra obtained for single cells in Fig. 5C. FRL cells acquire an additional emission band from Chl f at 735 nm (Fig. 5E right) which emit fluorescence at this wavelength appears to be distributed more evenly around the cell than the more peripheral 680-nm emission (Fig. 5, D and E, left). The emission signal at 735 nm will contain a contribution from FRL phycobiliproteins, which emit fluorescence at this wavelength in *Leptolyngbya* JSC-1 and *Synechococcus* 7335 (*59*). FRL phycobiliproteins potentially represent the dominant source of 735-nm emission in our spectral imaging, but the 485-nm excitation wavelength used heavily favors Chl a and Chl f over FRL-specific phycobiliproteins. The emission spectra from two individual FRL cells (denoted as cells 2 and 3 in Fig. 5B) have two maxima, at 680 and 735 nm, but the relative intensity of the peak differs between the cells, highlighting the variability of the FRL acclimation at the single-cell level. The images in Fig. 5 (D and E) also reflect this cell-to-cell variability.

Simultaneously with the spectral imaging, fluorescence lifetime maps of the immobilized cells were recorded. The photon fluence for all lifetime measurements was about 1.0 × 10^14^ photons pulse^–1^ cm^– 2^, which is sufficiently low to minimize excitonic annihilation in the antenna. As with spectral imaging, we collected 300 to 350 data points per cell; transient fluorescence decays were limited by the instrument response of the system, which is approximately 130 ps. A double-exponent amplitude-averaged lifetime image of WL cells, recorded at 680 nm, is shown in Fig. 5F; the distribution of the amplitude-averaged lifetimes across the image displayed in the histogram in Fig. 5I shows lifetime values in the range 800 to 1600 ps with a mean and an SD of 1315 ± 121 ps. Longer lifetimes (orange/red) tend to be found at the cell periphery in WL cells, following the same distribution for 680-nm emission seen in Fig. 5D (left). Lifetime images of FRL cells recorded at emission wavelengths of 680 and 735 nm with excitation from a 485-nm pulsed laser (Fig. 5, G and H) show markedly different lifetimes, with the mean and SD of 1090 ± 93 ps and 964 ± 55 ps, respectively (Fig. 5, J and K), where there is almost no amplitude in WL cells. Thus, acclimation to FRL involves not only the assembly of an antenna containing Chl a and Chl f but also FRL phycobiliproteins (see above) that tend to be coupled to a population of faster traps than found in WL. Longer lifetimes, associated with 680-nm emission (Fig. 5G), tend to be found in more peripheral regions of both WL and FRL cells (Fig. 5, F and G), whereas somewhat shorter lifetimes follow the pattern of Chl f emission in Fig. 5E and have a more uniform distribution (Fig. 5H).

Last, negatively stained thin sections of WL (Fig. 5, L and M) and FRL (Fig. 5, N and O) cells show that the FaRLiP response involves closer packing of thylakoid membranes likely because of the more compact phycobiliprotein complexes that are synthesized by FRL cells. The large, membrane-extrinsic hemidiscoidal phycobilisomes synthesized under WL normally impose separations of ~100 nm between thylakoids, as seen in Fig. 5M. Although the structure of the phycobiliprotein complexes assembled under FRL acclimation in *C. thermalis* 7203 has yet to be determined, they are likely to be smaller than those synthesized under WL conditions, which allow a closer association of adjacent thylakoid membranes (*25*, *59*–*62*), consistent with the images in Fig. 5 (N and O).

## DISCUSSION

### Altered configurations of PSI complexes in FaRLiP

The first structures of cyanobacterial PSI revealed a trimeric configuration (*53*, *63*), which is also the case in the model organism *Synechocystis* sp. PCC 6803 (hereafter *Synechocystis* 6803) (*17*). Other cyanobacteria, such as *Nostoc* 7120 (*46*, *47*, *50*, *51*, *64*) and *Chroococcidiopsis* sp. TS-821 (*48*, *49*), have dimeric and tetrameric PSI complexes, and there appears to be a correlation between heterocyst-forming cyanobacteria and the presence of monomeric, dimeric, and tetrameric, rather than trimeric, PSI complexes (*65*). Little was known about the arrangement of PSI in the thylakoids of FaRLiP cyanobacteria (*7*) or whether PSI organization undergoes dynamic changes in response to different wavelengths of light. In *C. thermalis* 7203 and *C. fritschii* 9212, we observed a marked change in the organization of PSI complexes in the thylakoid membranes between WL-grown and FRL-acclimated cells, with a non-trimeric arrangement seen in the former and a trimeric configuration in the latter. In the case of *Synechococcus* 7335, some trimeric complexes were observed in WL cells. However, there still appeared to be an increase in the proportion of trimeric PSI complexes in the thylakoid membranes in FRL-acclimated cells, consistent with the reorganization seen in *C. thermalis* 7203 and *C. fritschii* 9212. The trimeric arrangement of FRL-acclimated PSI is corroborated by recent cryo-EM structures of the complex (*29*, *30*).

The change from PSI dimers in WL to trimers in FRL did not affect the density of PSI complexes in the thylakoid membrane, with the number of PSI complexes per square micrometer remaining relatively constant. Pseudo-crystalline packing of trimeric and dimeric PSI complexes has previously been observed in thylakoid membranes in a number of cyanobacteria (*40*, *42*, *45*). This type of organization is hypothesized to be an adaptation to maximize the number of PSI complexes in the thylakoid membrane rather than to facilitate intercomplex energy transfer (*42*). Recently determined structures of PSI complexes from FRL-acclimated cyanobacteria (*29*, *30*) show that about six Chl a molecules are absent and another seven to eight Chl a molecules are replaced by Chl f, in agreement with estimations made in isolated PSI (*26*). In terms of membrane area, our AFM data show that there is a range of approximately 520,000 to 660,000 Chl a μm^−2^ in PSI domains of WL-grown cells for the three species studies here, which is altered by FRL acclimation to approximately 450,000 to 550,000 Chl a μm^−2^ and 38,000 to 46,000 Chl f μm^−2^. Thus, FRL acclimation adjusts the overall absorption of PSI but does not transform it. Mascoli *et al.* (*66*) present a detailed analysis of the advantages and compromises made when Chl f is incorporated into photosystems operating under FRL, and they conclude that Chl f confers important benefits on FaRLiP strains in environments that provide photons with wavelengths longer than 700 nm. Thus, despite the relatively small contribution made by Chl f to the overall absorption of photosystems, it still presents a large advantage over a purely Chl a–based photosystem lacking any capacity to absorb in the 720- to 750-nm region (*67*).

### Involvement of PsaL sequences in mediating the oligomeric state of PSI complexes

The changes in oligomeric state of PSI complexes upon FRL acclimation in all three organisms appear to be at least in part due to incorporation of the PsaL2 and PsaI2 PSI subunits encoded in the FaRLiP gene cluster. In other cyanobacteria, PsaL and PsaI are involved in mediating the interaction between constituent monomers of dimeric, trimeric, and tetrameric PSI complexes (*17*, *46*, *47*, *50*, *52*, *68*, *69*), and a previous study identified a specific motif in PsaL that seems to play a role in PSI oligomerization (*65*). Cyanobacteria with monomeric, dimeric, or tetrameric PSI, but not PSI trimers, frequently had a multiproline motif in the loop region of PsaL between the second and third transmembrane helices. This motif consisted of an NPPXP sequence followed by PNPP and was absent in most of the PsaL subunits in cyanobacteria where PSI is present as a trimer (*65*). The PsaL subunits encoded by the FaRLiP gene clusters of *C. thermalis* 7203 and *C. fritschii* 9212 do not contain this multiproline motif (fig. S6), consistent with the trimeric PSI complexes found under FRL growth conditions. In *C. fritschii* 9212, the PsaL subunit that is not encoded in the FaRLiP gene cluster has the full multiproline motif, and the non-FaRLiP–encoded PsaL subunit from *C. thermalis* 7203 has the initial NPPXP sequence buts lacks the following PNPP sequence (fig. S7). The presence of these multiproline motifs in the PsaL subunit expressed under WL conditions, and the lack of trimers observed in the AFM supports the hypothesis of Li *et al.* (*65*) that this motif plays a role in PSI oligomerization.

The situation with *Synechococcus* 7335 is slightly less clear; PsaL2, which is encoded within the FaRLiP gene cluster, does not contain the multiproline motif (fig. S7), and PSI trimers predominate in the FRL-acclimated cells, consistent with *C. thermalis* 7203 and *C. fritschii* 9212. However, the PsaL that is not encoded in the FaRLiP cluster also lacks the multiproline signature (fig. S7), yet PSI dimers are found in membranes from cells grown under WL. This suggests that, while the multiproline motif appears to play a role in preventing PSI trimer formation in some species, it is not essential for PSI dimerization or tetramerization and another mechanism must cause dimerization in *Synechococcus* 7335.

Malavath and co-workers (*17*) were able to identify several PsaL residues involved in the trimerization of the PSI complex; Q122, D149, R153, and N157 are all conserved in the PsaL2 subunits encoded within the FaRLiP gene clusters from all three organisms in this study (fig. S6). Conversely, these residues are absent from the PsaL1 subunits, which are not encoded by the FaRLiP gene cluster (fig. S7), consistent with the lack of trimer formation under WL growth conditions. Alignments of the PsaL sequences of the species used in this study were made with PsaL subunits from *Synechocystis* 6803 and *Nostoc* 7120, which form trimeric PSI and tetrameric PSI complexes, respectively (figs. S6 and S7). The PsaL1 subunits found under WL had greater sequence similarity with the *Nostoc* 7120 PsaL, whereas the FRL PsaL2 subunits were more similar to the *Synechocystis* 6803 protein. This is consistent with PSI complexes being non-trimeric under WL conditions and adopting a trimeric configuration during FRL growth.

Deletion of the *psaI* and *psaL* genes of *Synechococcus* sp. PCC 7002 caused partial or complete loss of PSI trimers and destabilization of PsaM binding (*69*). The PsaI subunit, a small polypeptide with a single transmembrane helix, was also shown to play a role in PSI trimerization in *Synechocystis* 6803, with its D2 residue interacting with R153 of the adjacent PsaL subunit (*17*). The PsaI2 subunits encoded in the FaRLiP gene cluster have an aspartate residue at position 3 in the polypeptide chain as part of a conserved motif with the sequence MVDMTQL*G, whereas the PsaI1 subunits of WL-PSI lack an N-terminal aspartate (fig. S8). This further suggests that incorporation of the subunits encoded by the FaRLiP gene cluster into the PSI structure is responsible for the reorganization of PSI complexes observed in this study.

### FRL-specific function of IsiA-like Chro_2988 and Chro_2989 proteins

FaRLiP leads to the synthesis of alternative paralogs of PSI subunits that at least partially control the oligomeric state of PSI imaged by AFM in Figs. 1 to 3. However, a protein designated as Chro_2988 was 80-fold less abundant in thylakoid membranes from FRL-acclimated cells of *C. thermalis* 7203 compared to WL-grown samples. Thus, the assembly of PSI trimers in FRL could be linked to the greatly diminished levels of Chro_2988, which has a domain with homology to PsaL and a domain with homology to the Chl a–binding protein, IsiA. The PsaL domain of Chro_2988 has a greater sequence similarity with the non-FaRLiP–encoded PsaL protein and has part of the multiproline motif that is associated with the PsaL subunits found in non-trimeric PSI complexes (fig. S7). It also lacks the residues identified as being important for trimer formation (*17*), suggesting that, in WL, the PsaL domain of Chro_2988 could disrupt the interaction between PsaL subunits and prevent trimer formation. The 80-fold reduction in the level of Chro_2988 in FRL-acclimated membranes would allow the PsaL proteins of monomeric PSI to interact, leading to formation of the trimeric PSI complexes observed in the AFM data. A paralog of the Chro_2988 protein is present in *C. fritschii* 9212 but not in *Synechococcus* 7335, which could account for the formation of an organized PSI lattice in WL *Synechococcus* 7335. The similarly disordered appearance of PSI complexes in WL-grown *C. fritschii* 9212 and *C. thermalis* 7203 could be a consequence of Chro_2988 binding to PSI subunits and disrupting lattice formation.

The other membrane-intrinsic protein that displayed a marked change in abundance between WL and FRL growth conditions was Chro_2989, a paralog of the IsiA protein. It is considered to be a paralog of IsiA rather than CP43 because it lacks the characteristic “E loop” of CP43 proteins, which is absent from IsiA and Pcb proteins (*70*). The ~80-fold decrease in abundance of this protein between WL and FRL mirrors that of Chro_2988, suggesting a functional relationship between them. It is possible that Chro_2988 and Chro_2989 form a heterodimer through a similar interaction that allows IsiA complexes to form oligomeric structures around photosystems. Such a heterodimer could interact with the PsaL subunit of PSI through the PsaL domain of Chro_2988. While there is no direct evidence to show that this is the case, it has been shown that a Chro_2988 paralog is able to associate with other IsiA paralogs to form supercomplexes under iron starvation conditions in *Leptolyngbya* sp. JSC-1 (*56*). Chro_2988-type proteins may have widespread importance, as paralogs are found in other known/predicted FaRLiP cyanobacteria, including *Leptolyngbya* sp. JSC-1, *Calothrix* sp. PCC 7507, *F. thermalis* 7521, *C. fritschii* PCC 6912, *Pleurocapsa* sp. PCC 7327, and *Oscillatoriales* sp. JSC-12.

### Remodeling of phycobilisomes under FRL acclimation

Phycobiliproteins encoded by the FaRLiP gene cluster have absorption maxima at >700 nm, which increases the capacity of cyanobacteria to harvest FRL (*7*, *25*, *39*, *59*, *60*). It has also been shown that there is variation in the assembly of FRL-absorbing phycobiliproteins into antenna complexes in different cyanobacteria capable of FaRLiP. Three distinct phycobilisome responses have been characterized as part of FaRLiP in three different cyanobacteria. *Leptolyngbya* sp. JSC-1 assembles hemidiscoidal phycobilisomes containing pentacylindrical allophycocyanin (APC) cores in WL and RL, which are remodeled under FRL acclimation into phycobiliproteins that contain pentacylindrical and bicylindrical cores. These structures comprise FaRLiP gene cluster–encoded red-shifted APC subunits attached to rods containing phycocyanin (PC) and phycoerythrin (PE) (*7*, *59*). A recent study of FRL phycobiliproteins from *Leptolyngbya* JSC-1 and *Synechococcus* 7335 produced recombinantly in *Escherichia coli* showed that their red-shifted absorption largely arises from two differences (*59*). The phycocyanobilin chromophore on the α subunits of FRL-absorbing APCs has a more planar conformation and, after oligomerization, there are excitonic interactions with the chromophore on the β subunit of an adjacent protomer. The former interactions do not occur in the α subunits of paralogous APCs that absorb orange-red light (*59*, *62*). Remodeling of phycobilisomes is more marked in *H. hongdechloris* during FaRLiP, with these large antenna assemblies downsized to a bicylindrical core, two side-by-side cylinders of APC trimers lacking any PC or PE rods (*60*). FaRLiP in *Synechococcus* 7335 involves another type of phycobilisome reorganization, with the assembly of novel bicylindrical phycobiliprotein complexes containing FRL-absorbing APCs that lack PC or PE rods. In contrast to the previous two examples, the original tricylindrical phycobilisomes are not remodeled or degraded, and a population of these phycobilisomes persists for months after initial acclimation to FRL (*25*, *59*); this is likely to enable rapid utilization of WL should it become available in the short term.

In the case of *C. thermalis* 7203, the FaRLiP response of the phycobilisomes appears to differ from those in *Leptolyngbya* sp. JSC-1, *H. hongdechloris*, and *Synechococcus* 7335, although there are some similarities. The phycobiliprotein paralogs encoded in the FaRLiP gene cluster are accumulated either exclusively under FRL acclimation in the case of ApcD2, ApcD3, ApcD5, and ApcE2, or at approximately 1000 times the levels of WL-grown cells as seen with ApcB2, which enables *C. thermalis* 7203 to absorb wavelengths of light >700 nm (*71*, *72*). The EM thin sections of *C. thermalis* 7203 in Fig. 5 show that the thylakoid membranes are more densely packed in the FRL-acclimated cells, with less space between the parallel sheets of thylakoid membrane, as also seen for *H. hongdechloris*, for example (*61*). This would imply that the smaller phycobilisomes, relative to those produced under WL, allow a closer approach between the cytoplasmic surfaces of adjacent thylakoid membranes. The distance between adjacent thylakoid membranes was also reduced in *Synechocystis* 6803 mutants in which the phycobilisomes are either truncated or completely absent (*73*). This would parallel the response seen in *Synechococcus* 7335, *H. hongdechloris*, and *Leptolyngbya* sp. JSC-1, which have phycobiliprotein antenna assemblies that are much smaller in size under FRL acclimation. Despite having very similar FaRLiP gene clusters, there appears to be marked divergence in the phycobilisome response in different FaRLiP cyanobacteria when grown under FRL. Further work is required to purify phycobilisomes to fully characterize the changes in phycobilisome assembly and distribution in *C. thermalis* 7203 to augment recent recombinant, modeling, and spectroscopic studies on FaRLiP in *Leptolyngbya* JSC-1 and *Synechococcus* 7335 (*25*, *45*, *59*, *62*).

### Other intrinsic membrane proteins involved in FaRLiP

The FaRLiP cluster contains the *chlF* gene that encodes the membrane-bound light-dependent Chl f synthase, ChlF (*24*, *36*). Although Chl f was found in FRL membrane samples from all three cyanobacteria examined in this study, *C. thermalis* 7203, *Synechococcus* 7335, and *C. fritschii* 9212 (fig. S9), ChlF was not detected in the proteome of either WL or FRL membranes of *C. thermalis* 7203. Similarly, the Chl a synthase (ChlG) is often undetected in mass spectrometric analysis of cyanobacterial proteomes. For example, in 32 quantitative proteomics studies in *Synechocystis* 6803, ChlG was only detected in 9 of them; even in analyses of purified membrane fractions only two of seven proteomes identified ChlG, likely because of the low abundance of this integral membrane protein (*74*, *75*). In a recent proteomic analysis in which approximately 50% of the theoretical proteome was detected, including 12 of the Chl biosynthesis pathway components, it was still not possible to detect ChlG (*76*). ChlF appears to be similarly elusive, and further work will be required to quantify this protein.

Another important change in the proteome associated with FaRLiP was the lowered levels of the ATP synthase and proteins associated with photosynthetic electron transport. These changes are consistent with an acclimation to FRL; for example, our *C. thermalis* 7203 cultures were grown using a 750-nm light source with a 22-nm full width at half maximum, and although Chl f is necessary to extend absorption above 700 nm to make use of this source of energy (*66*), this pigment represents only ~10% of the total Chl, with the remainder as Chl a (*26*). Overall, the energy input relative to cells grown in WL is greatly diminished under FRL growth conditions, and the lower levels of electron transport and ATP synthase components observed here are a manifestation of FRL growth as a low-light condition in which less photosynthetic electron transport takes place. It is possible that the slower charge separation recently reported in FRL-PSII (*66*, *77*) entails lower levels of downstream electron transport or ATP synthase components than those required in WL. There is an interesting parallel with the effect of low light on the relative levels of photosynthetic electron transport components in *Arabidopsis*; a lowered flux of electrons and lower levels of ATP synthesis corresponded to diminished levels of cytochrome *b*_6_*f*, NDH-1, and ATP synthase (*78*).

### Fluorescence imaging of FRL-acclimated cells

Spectral and fluorescence lifetime imaging of individual WL and FRL cells of *C. thermalis* 7203 highlights the cell-to-cell variability of the FaRLiP response and hints at an altered cellular distribution of fluorescing complexes. Excitation of samples at 485 nm is weakly absorbed by Chls and more strongly so by photosystem carotenoids; in addition, it elicits fluorescence from the abundant phycobilisomes, which could contribute to the relatively long-lived (1315 ± 121 ps) 680-nm emission from the peripheral regions of WL cells, seen also in FRL cells. Shorter lifetimes (964 ± 55 ps) from 735-nm emitters were distributed evenly within the FRL cells. The presence of red-shifted phycobiliproteins in FRL cells (*45*, *59*, *62*) likely adds another component to these lifetimes. Our FLIM setup was configured for spatial measurements, with between 300 and 350 data points per cell, but lifetime components shorter than 130 ps are not resolved. In contrast, the time-resolved fluorescence study of Mascoli and co-workers (*66*) used 8- to 90-ps time resolutions to examine energy trapping in ensembles of complexes, membranes, and whole cells of WL-grown and FRL-acclimated *C. fritschii* 9212 and *C. thermalis* 7203. They obtained an average lifetime of 300 ps for 680-nm emission from WL *C. thermalis* 7203 cells and 900 ps for 740-nm emission from FRL cells, in each case with PSII in a closed state, reflecting a general slowing of energy trapping in FRL. Our FLIM measurements of 735-nm emission from FRL cells likely include a ~150-ps contribution from red-shifted FRL phycobiliproteins coupled to PSI (*66*), the lifetimes of which we cannot resolve directly but are still reflected in Fig. 5K as a skewed distribution of lifetimes to shorter values. AFM of *C. thermalis* 7203 thylakoids shows that these changes in the cellular distribution of fluorescence lifetimes are accompanied by remodeling of PSI dimers in WL to trimers in FRL and a structural rearrangement of PSI domains. A similar rearrangement is seen for *Synechococcus* 7335 in WL/FRL (Fig. 3) and RL/FRL (*45*), which has been suggested to accommodate an alteration in the size of phycobilisomes in areas of the thylakoid where PSI and PSII can interact and form megacomplexes (*45*).

In conclusion, we have shown the effect of substitution of PSI subunits with paralogous proteins during FaRLiP (*7*) on the supramolecular assembly of this photosystem at the membrane and cellular levels. The change in PSI from a non-trimeric state in WL to a trimeric state in FRL was consistent in three species of cyanobacteria. In the case of *C. thermalis* 7203, proteomic analysis indicates that interactions between FRL-specific PSI subunits may orchestrate the observed restructuring, with consequent effects on energy transfer and trapping in individual cells. These approaches can be combined with future studies exploring the effects of incorporating paralogous PSII subunits, and the assembly and association of FRL-remodeled phycobilisomes with the thylakoid membrane, to give a more complete picture of how cyanobacteria adapt to use FRL for photosynthesis.

## MATERIALS AND METHODS

### Cyanobacterial strain and growth conditions

Cells of *C. thermalis* PCC 7203 were grown in liquid BG11 medium (*79*) at 30°C to an optical density at 780 nm = 0.4 to 0.6. Cells were cultured in 10-liter bottles bubbled with sterile air and stirred every 24 hours. The light conditions used were WL of ~30 μmol of photons m^−2^ s^−1^ (Quantitherm light meter, Hansatech) or FRL (750 nm; Epitex, L750-01AU) of ~30 μmol of photons m^−2^ s^−1^ (optical power meter, Newport). *Synechococcus* 7335 was grown in a modified ASN III growth medium, and *C. fritschii* PCC 9212 was grown in B-Hepes medium as previously described (*24*). WL was provided by cool white fluorescent tubes at ~250 μmol of photons m^−2^ s^−1^ for *C. fritschii* 9212 and ~50 μmol of photons m^−2^ s^−1^ for *Synechococcus* 7335. FRL was provided by LEDs (Marubeni, Santa Clara, CA) with emission centered at 720 nm in combination with plastic filters at an intensity of approximately 26 to 30 μmol of photons m^−2^ s^−1^ (*24*, *36*).

### Preparation of crude membranes

Cells were resuspended in buffer containing 25 mM potassium phosphate (pH 7.4), 100 mM NaCl, and 10 mM MgCl_2_; 1 liter of cells was resuspended in approximately 10 ml of buffer. A volume of 1 ml was mixed with the same volume of 0.1-mm glass beads and broken by eight rounds of 30 s of bead beating in a Mini-BeadBeater (BioSpec Products). A sucrose step gradient composed of 9.5 ml of 30% (w/w) sucrose on a 2.0-ml 50% (w/w) sucrose cushion was poured in a SW41 centrifuge tube; sucrose solutions were made up in a buffer containing 25 mM potassium phosphate (pH 7.4), 100 mM NaCl, and 10 mM MgCl_2_. The 1-ml cell lysate was added to the top of the sucrose gradient, which was centrifuged at 110,000*g* at 4°C for 30 min. A green band at the interface between the 30 and 50% sucrose solutions containing the thylakoid membranes was harvested and either used immediately or flash-frozen with liquid nitrogen and stored at −80°C for later use.

### Preparation of membranes for imaging by AFM

Crude membranes (1 ml) were applied atop a 11.5-ml continuous sucrose gradient made from equal volumes of 20 and 50% (w/w) sucrose with 0.1% (w/w) digitonin. This gradient was centrifuged at 200,000*g* at 4°C for 2 hours. Thylakoid membranes were present as a green smear that ran approximately the length of the gradient; samples were taken from a restricted region of each gradient, delineated by the dashed lines in Figs. 1 to 3 (A and B), and a gallery of membrane patches harvested from each location is shown in figs. S10 to S12.

### AFM imaging

A MultiMode 8 AFM with a Nanoscope 8.0 controller (Bruker Nano Surfaces) was used to image thylakoid membrane samples. Approximately 5 μl of membrane sample was applied to a freshly cleaved mica disc along with 45 μl of AFM buffer [10 mM Hepes (pH 7.5) and 100 mM KCl]. After approximately 1 hour, the mica was washed three times with 50 μl of the same buffer. The final wash was left on the surface of the mica disc, which was then mounted into the AFM scanner (J-scanner). Samples were imaged using a SNL AFM probe (Bruker Nano Surfaces) in an MTFML fluid cell (Bruker Nano Surfaces) using the PeakForce Nanomechanical Mapping mode under liquid. Once the probe was loaded into the fluid cell, the reservoirs were filled with buffer containing AFM buffer, the fluid cell was mounted onto the AFM, and the laser was aligned with the probe. Images were taken at 256 × 256 or 512 × 512 samples per line using a Peak Force amplitude of 5 to 20 nm with the force imparted on the sample varying between 5 and 1000 pN. Image processing and analysis were performed using NanoScope Analysis 1.9 or Gwyddion v.2.52 (*80*).

### Proteomic analysis of thylakoid membranes

Thylakoid membranes in 25 mM potassium phosphate (pH 7.2) and 100 mM NaCl were pelleted by centrifugation at 16,600*g* and then solubilized by incubation in 50 μl of 2% (w/v) SDS and 60 mM dithiothreitol at 95°C for 5 min. The proteins were extracted by precipitation using a 2-D Clean-Up kit (GE Healthcare) following the manufacturer’s instructions, and the pellets were dissolved in a minimal volume of 8 M urea and 100 mM tris-HCl (pH 8.5), with protein concentration determined by NanoDrop at 280 nm. Fifty micrograms of protein was subjected to reduction and S-alkylation with 5 mM tris(2-carboxyethyl)phosphine-HCl (37°C for 30 min) and 10 mM iodoacetamide (room temperature, in the dark, for 30 min), respectively. Proteolytic digestion with a combination of endoproteinase Lys-C and trypsin and analysis by nanoflow liquid chromatography coupled to mass spectrometry (Q Exactive HF, Thermo Fisher Scientific) were performed as described previously (*42*, *81*). Protein identification by database searching (*C. thermalis* 7203 reference proteome, 5740 proteins, downloaded from www.uniprot.org/proteomes/UP000010384 on 9 January 2018) in combination with label-free quantification by the iBAQ (*54*) and Top-N (*58*) methods was performed by MaxQuant v. 1.5.3.30 (*82*). Processing of quantification results, including normalization to the intra-analysis sum of protein abundance scores (iBAQ) or peptide ion intensities (Top-N) (*83*) and statistical analysis, was performed using Perseus v. 1.5.3.2 (*84*).

### Fluorescence lifetime imaging

Live cells were spotted on agarose gel, mounted on a microscope slide, and covered with a coverslip, thus keeping the cells under near-physiological conditions. Fluorescence lifetime imaging was performed on a home-built lifetime imaging microscope equipped with a spectrometer (Princeton Instruments, Acton SP2558), electron-multiplying charge-coupled device (CCD) camera (Princeton Instruments, ProEM 512), and a single-photon hybrid photodetector (Becker & Hickl, HPM-100-50). The microscope is equipped with two different excitation light sources: a 470-nm LED light (Thorlabs, M470L2) for widefield fluorescence images and a 485-nm pulsed laser (PicoQuant, LDH-D-C-485) with variable repetition rate for spectral and lifetime imaging. The photon fluence for all lifetime measurements was about 1 × 10^14^ photons pulse^–1^ cm^–2^. Fluorescence emission detection was filtered through a 605-nm dichroic mirror (Semrock, FF605-Di02) and either a 647-nm long-pass filter (Semrock, BLP01-647R-25) for the acquisition of the spectral data, or a 679/41-nm bandpass filter (Semrock, FF01-679/41-25) and a 725/40-nm bandpass filter (Semrock, FF01-725/40-25) for the time-resolved data acquired from WL and FRL cells, respectively. A secondary slit in front of the single-photon detector allowed further spectral narrowing of the measured signal; typically, we were able to select ±6 nm around the central wavelength selected by the monochromator and matched to the peak emission wavelength of the sample. The laser beam was focused on the sample surface to a diffraction limited spot using a 100× objective (PlanFluorite, numerical aperture = 1.4, oil immersion, Olympus), and the modulation of the laser was synchronized with a time-correlated single-photon counting module (Becker & Hickl, SPC-150). The spatial resolution of our instrument is comparable with that of a commercial confocal laser scanning microscope and is determined by the size of the focused laser spot on the sample surface (typically around 250 to 300 nm). The pixel size in the spectral and lifetime images is 125 nm, which results in 300 to 350 data points per cell, depending on cell size. During the lifetime data analysis, we used 3 × 3 pixel binning to take into account the illumination of the neighboring pixels by the laser (the illumination spot covers roughly 3 × 3 pixels of the image). Since the binning is done for each pixel of the image, the analyzed lifetime data still contain the full number of data points per cell. FLIM images were analyzed with OriginPro and FLIMfit software packages by fitting a multiexponential decay function (Eq. 1)

$$I(t)=\sum_{i=1}^{n}A_{i}\text{exp}(\frac{-t}{\tau_{i}})+B$$ (1)

where τ_i_ is the fluorescence lifetime, *A_i_* is the fractional amplitude contribution of the *i*th decay component, and *B* is the background. The quality of fit was judged on the basis of the reduced χ^2^ (Eq. 2)

$${\chi^{2}}_{\text{red}}=\frac{\sum_{k=1}^{n}\frac{{\left[I(t_{k})-I_{c}(t_{k})\right]}^{2}}{I(t_{k})}}{n-p}=\frac{\chi^{2}}{n-p}$$ (2)

where *I(t_k_*) is the data at time point *k*, *I_c_(t_k_)* is the fit at time point *k*, *n* is the number of data points, and *p* is the number of variable fit parameters (*n* – *p* = degrees of freedom). The instrument response function (IRF) of the system, measured using a mirror instead of a sample, was ~130 ps, and the convolution of the decay curves with the IRF was accounted for during fitting.

### Pigment analysis

Proteins of thylakoid membrane were pelleted by centrifugation at 200,000*g* at 4°C for 1 hour. Pigments were extracted from cell pellets in 100 μl of methanol with vortexing at room temperature under dim green light. Samples were centrifuged at 21,000*g* at 4°C for 15 min to remove insoluble material, and 80 μl of the supernatant was analyzed by reversed-phase high-performance liquid chromatography (RP-HPLC) using an Agilent 1200 HPLC system equipped with a Discovery HS C18 5-μm column (column dimensions, 25 cm by 4.6 mm). The column was pre-equilibrated in 35:65 solvent A to solvent B, where solvent A was 350 mM ammonium acetate/30% (v/v) methanol and solvent B was 100% methanol. A flow rate of 1 ml min^−1^ was used to separate pigments using a linear gradient from 65 to 100% solvent B over a 15-min period, followed by isocratic elution with 100% solvent B for 17 min. The column was re-equilibrated with 35:65 solvent A to solvent B for 7 min before analysis of the next sample. Elution of Chl a and Chl f was monitored by absorption at 665 and 707 nm, respectively, and each pigment was identified by its characteristic absorbance spectra.

### Transmission electron microscopy

Proteins used for TEM (fig. S1 and S2) were adjusted to a Chl concentration of ~0.7 mg of Chl ml^−1^, and 4 μl was applied to a glow-discharged (30 s, 25 mA) microscopy grid (Electron Microscopy Sciences; 400-mesh copper with 5- to 6-nm Formvar and 3- to 4-nm carbon). After 30 s of incubation, excess liquid was wicked from the grid using a piece of filter paper and washed three times in a uranyl acetate solution (2%, w/v), wicking liquid between washes with filter paper. Grids were imaged on an FEI Tecnai F20 electron microscope at 200 kV equipped with a Gatan CCD camera. Samples for thin section EM were prepared as described previously [*85*; for a detailed protocol, see (*86*)].

### BN-PAGE analysis

For BN-PAGE analysis of *C. thermalis* 7203, thylakoid membranes were isolated as follows. Frozen cell pellets were resuspended in 50 mM MES-NaOH (pH 6.5), 5 mM CaCl_2_, 10 mM MgCl_2_, and 0.5 M sorbitol in the presence of protease inhibitors (0.6 mM aminocaproic acid, 0.6 mM benzamidine, and 1.25 mM bovine serum albumin). A spatula of deoxyribonuclease I was added before cell breakage using a CF Cell Disrupter (Constant Systems Ltd.). Cells were broken by two passages at ~200 MPa and unbroken cell material was removed by centrifugation at 10,000*g* and 4°C for 7 min. The supernatant was transferred to a fresh tube, and thylakoid membranes were sedimented by centrifugation at 193,000*g* at 4°C for 30 min. Membranes were resuspended in 50 mM MES-NaOH (pH 6.5), 5 mM CaCl_2_, and 10 mM MgCl_2_, centrifuged as previously described and washed two times to remove phycobilisomes. After the final centrifugation step, thylakoid membranes were resuspended in 50 mM MES-NaOH (pH 6.5), 5 mM CaCl_2_, 10 mM MgCl_2_, and 12.5% (v/v) glycerol, frozen in liquid nitrogen, and stored at −80°C. For membrane solubilization, the Chl concentration of the thylakoid membranes was adjusted to 0.2 mg ml^−1^, and *n*-dodecyl-β-d-maltoside (DDM) was added at a concentration of 0.4 or 2% (w/v), respectively. The samples were incubated for 1.5 hour at 4°C and centrifuged (30 min at 4°C and 22,000*g*). The photosystem containing supernatants were separated on 4 to 16% NativePAGE gels (Invitrogen) using the method described (*87*).

### Sucrose gradient fractionation of photosystems

Cells grown under different light conditions were harvested and resuspended in 50 mM tris-HCl (pH 8.0) buffer. After homogenization, cells were broken by three passages through a chilled French pressure cell at 138 MPa. After removing unbroken cells and larger debris by centrifugation, thylakoid membranes were pelleted by ultracentrifugation at 125,800*g* for 1 hour. Membranes were resuspended, adjusted with 50 mM tris-HCl (pH 8.0) buffer to ~0.4 to 0.5 mg of Chl ml^−1^, and solubilized by addition of 1% (w/v) DDM at 4°C for 1 hour. The resulting solution was centrifuged at 22,000*g* for 10 min to remove insoluble material. The supernatant was then loaded onto 5 to 20% (w/v) sucrose gradients containing 0.05% (w/v) DDM, which were centrifuged at 140,000*g* for 12 to 16 hours at 4°C (*7*, *28*).
