# Supplementary material for: Changes in supramolecular organization of cyanobacterial thylakoid membrane complexes in response to far-red light photoacclimation
Source: Sci Adv. 2022 Feb 9;8(6):eabj4437. doi: 10.1126/sciadv.abj4437 (PMC8827656; doi:10.1126/sciadv.abj4437)
Supplement: Supplementary file 1 — Figs. S1 to S12 Legends for Data S1 and S2 [file sciadv.abj4437_sm.pdf]

Science Advances
AAAS

Supplementary Materials for

Changes in supramolecular organization of cyanobacterial thylakoid membrane complexes in response to far-red light photoacclimation

Craig MacGregor-Chatwin, Dennis J. Nürnberg, Philip J. Jackson,
Cvetelin Vasilev, Andrew Hitchcock, Ming-Yang Ho, Gaozhong Shen, Christopher J. Gisriel,
William H. J. Wood, Moontaha Mahbub, Vera M. Selinger, Matthew P. Johnson,
Mark J. Dickman, Alfred William Rutherford, Donald A. Bryant, C. Neil Hunter*

*Corresponding author. Email: c.n.hunter@sheffield.ac.uk

Published 9 February 2022, *Sci. Adv.* **8**, eabj4437 (2022)
DOI: 10.1126/sciadv.abj4437

The PDF file includes:

Figs. S1 to S12
Legends for Data S1 and S2

Other Supplementary Material for this manuscript includes the following:

Data S1 to S2

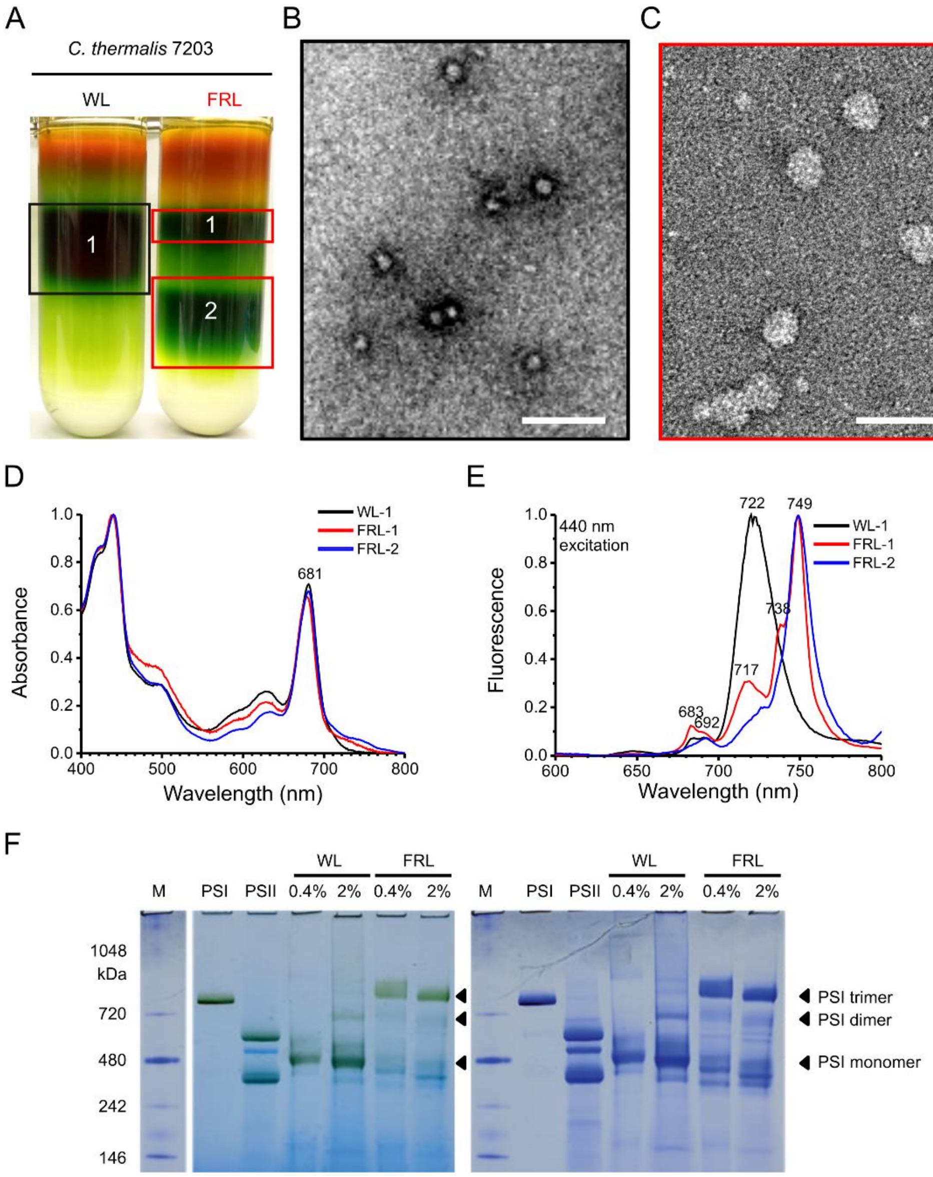

Fig. S1. Characterisation of photosystem complexes solubilised from thylakoid membranes from WL- and FRL-acclimated *C. thermalis* 7203. (**A**) Sucrose density gradients of solubilised membranes from WL (left) and FRL (right) cells showing green bands that correspond to different oligomeric states as described in the main text. (**B**) Negative stain transmission EM of the green band highlighted by the black rectangle "1" in (**A**) showing either monomeric or dimeric complexes. (**C**) Negative stain transmission EM of the band highlighted in the red rectangle "2" in (**A**) showing trimeric PSI complexes purified from FRL membranes. Scale bars in (**B**) and (**C**) are 50 nm. (**D**) Room temperature absorbance spectra of bands from the sucrose gradients in (**A**). (**E**) 77 K fluorescence emission spectra of bands from the sucrose gradients in (**A**). (**F**) BN-PAGE analysis of thylakoid membranes from WL- and FRL-acclimated *C. thermalis* 7203 (left, unstained gel; right, Coomassie-stained gel). Thylakoid membranes were solubilized in 0.4 % and 2 % (w/v) β-DDM as indicated. Isolated photosystems from *T. elongatus* were used for comparison, showing trimeric PSI and monomeric and dimeric PSII with an unknown protein complex in between. NativeMARK Protein Standard (Novex) was used as a molecular weight marker (M). Approximately 1.5 µg Chl was loaded for each condition.

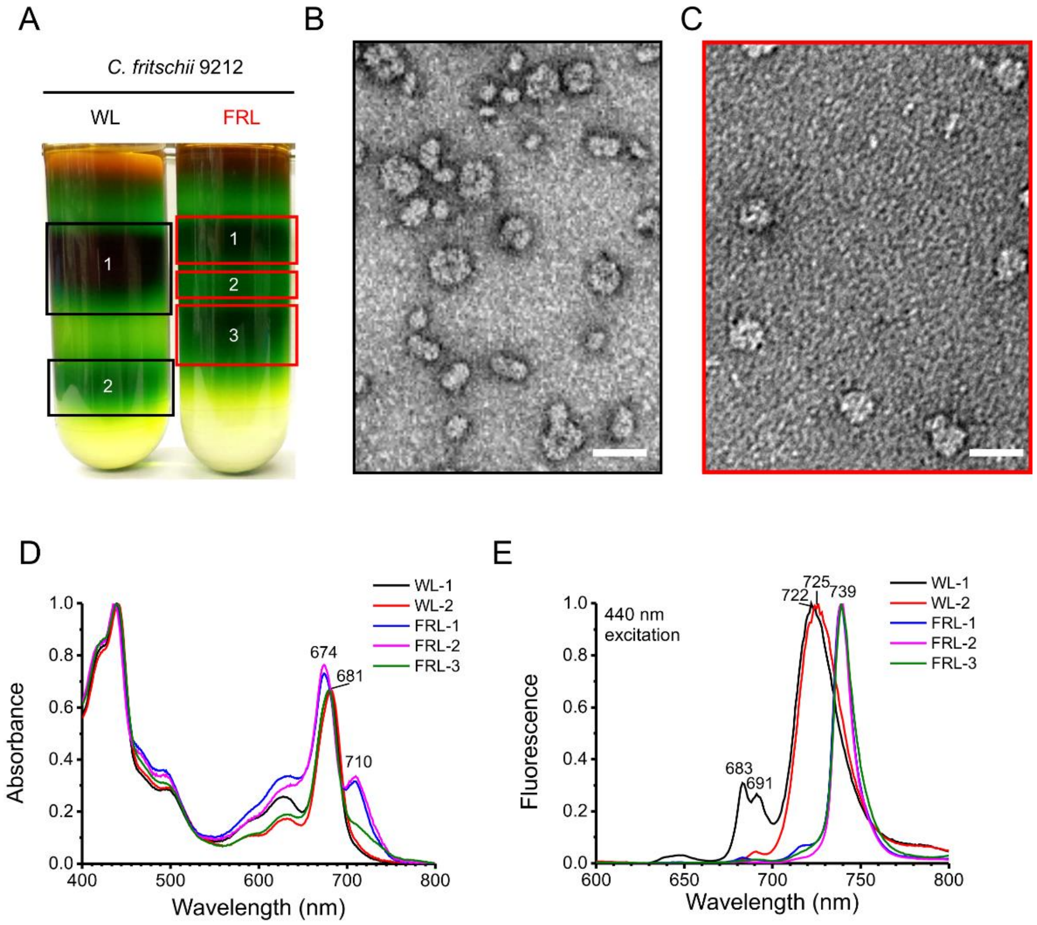

Fig. S2. Characterisation of photosystem complexes solubilised from thylakoid membranes from WL- and FRL-acclimated *C. fritschii* 9212. (**A**) Sucrose density gradients of solubilised membranes from WL (left) and FRL (right) cells showing green bands that correspond to different oligomeric states as described in the main text. (**B**) Negative stain transmission EM of the green band highlighted by the black rectangle "2" in (**A**) showing either dimeric or tetrameric complexes from WL membranes. (**C**) Negative stain transmission EM of the band highlighted in the red rectangle "3" in (**A**) showing trimeric PSI complexes purified from FRL-acclimated membranes. Scale bars in (**B**) and (**C**) are 50 nm. (**D**) Room temperature absorbance spectrum of bands from the sucrose gradients in (**A**). (**E**) 77 K fluorescence emission spectra of bands from the sucrose gradients in (**A**).

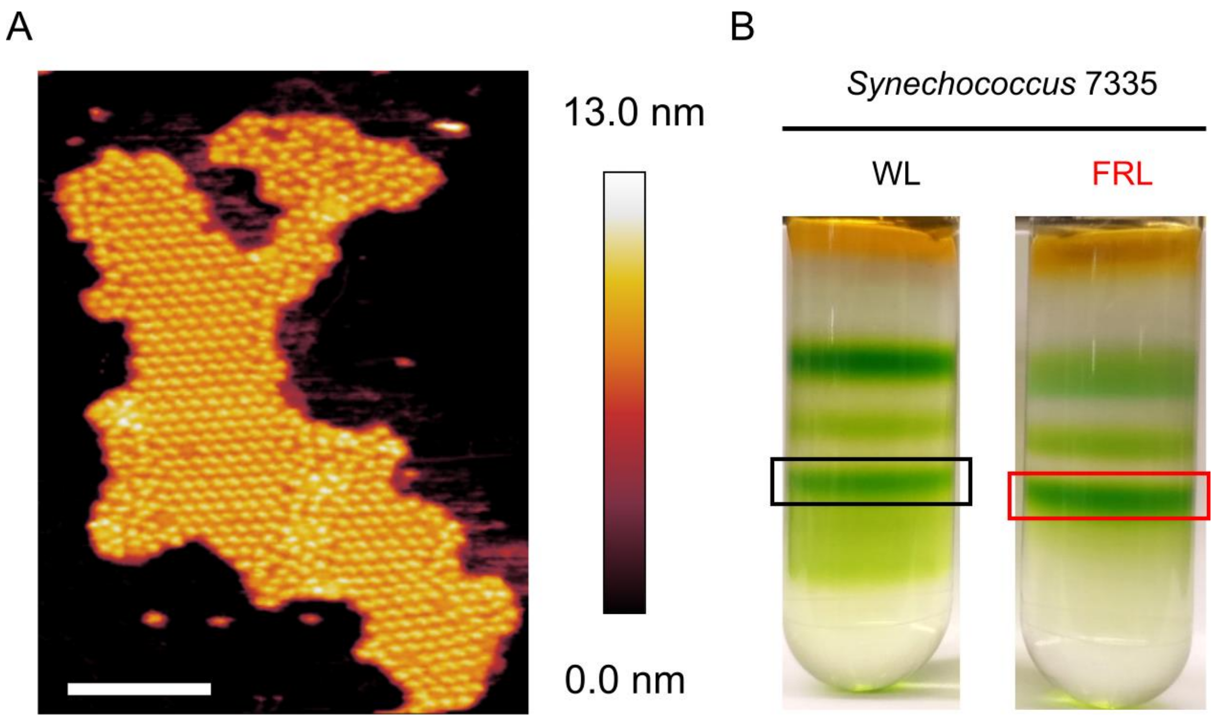

Fig. S3. Characterisation of PSI complexes in WL- and FRL-acclimated membranes from *Synechococcus* 7335. (**A**) AFM of thylakoid membranes from *Synechococcus* 7335 showing large arrays of PSI complexes in either a dimeric or tetrameric configuration with peripheral regions of trimeric PSI complexes in a disordered state, showing that both configurations are present in WL membranes. Scale bar is 100 nm.. (**B**) Sucrose density gradients of solubilised membranes from WL (left) and FRL (right) cells in which green bands can be observed that correspond to different oligomeric states of photosynthetic complexes. The black rectangle highlights the green band that corresponds to trimeric PSI in WL membranes. The red rectangle highlights the major band in the density gradient, which corresponds to PSI trimers, and suggests an increased proportion of trimeric PSI complexes in FRL-acclimated membranes.

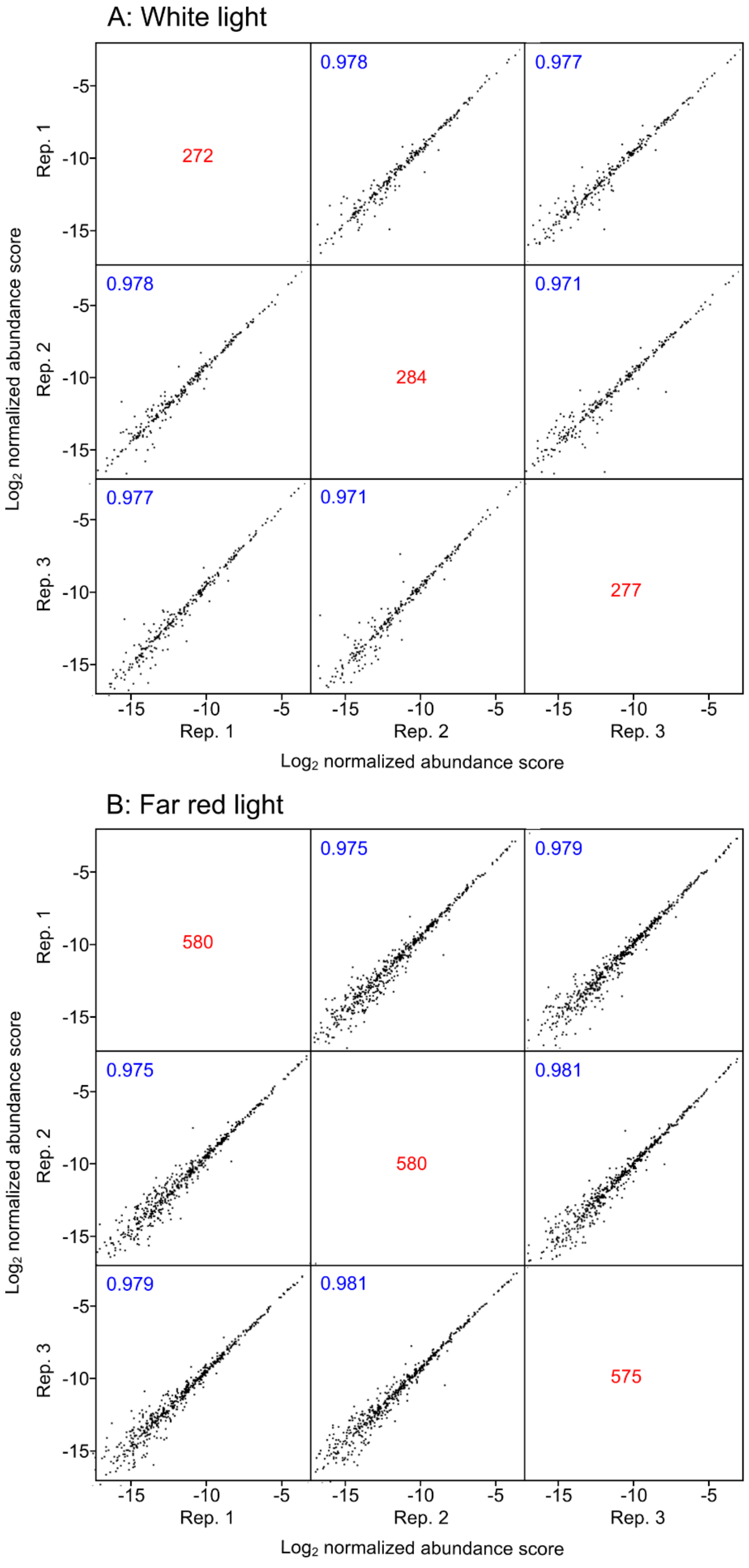
A: White light
272
284
277
0.978
0.977
0.971
Rep. 1
Rep. 2
Rep. 3
-5
-10
-15
Log2 normalized abundance score
B: Far red light
580
580
575
0.975
0.979
0.981

Fig. S4. Correlation matrices for three technical replicate proteomic analyses of thylakoid membranes from *C. thermalis* cells acclimated under (A) WL and (B) FRL. Thylakoid membranes were subjected to quantitative proteomic analysis in triplicate as described in Materials and Methods, with protein abundance scores calculated by the iBAQ method and normalized to the intra-analysis sum of iBAQ abundance scores. The numbers of protein identifications for each replicate are shown in red and the Spearman rank correlation coefficients in blue. A total of 629 proteins were quantified and the data-points are listed in Supplementary Data S1.

A

```
PsaA1         MTISPPEREEKKARVVVDNDPVPTSFELWSKPGHFDRTLSRGPKTTTWIWNLHALAHDFD     60
PsaA2         MTITP--EREQKVRVVVDNDPVPTSPELWAKPGHFDRTLARGPKTTTWIWNLHANAHDFD     58

PsaA1         THTSDLEDISRKIFAAHFGHLAVIFIWLSGMYFHGARFSNYEAWLADPLGVKPSAQVVWS     120
PsaA2         THTSDLEDISRKIFAAHFGHLAVIFIWLSGMYFHGAKFSNFEAWMANPTGVKPSAQVVWS     118

PsaA1         VVGQDILNADVGGGFHGIQITSGFFQIWRGAGITNTFQLYCTAIGGLVMAALMLFAGWFH     180
PsaA2         LVGQDILNADVGGGFHGIQITSGLFQLWRAAGITNTFQLYCTAIGGLVMAAIMLFAGWFH     178

PsaA1         YHKRAPKLEWFQNVESMLNHHLAGLLGLGSLAWAGHQIHVSLPINKLLDAGVAPKDIPLP     240
PsaA2         YHKRAPKLEWFQNWEAMMNHHLAGLLGLGCLGWAGHQIHVALPVNKLLDAGVAIKDIPLP     238

PsaA1         QEFILNSNLMTELYPSFAQGLTPFWTLNWGAYADFLTFKGGLNPVTGGLWLTDQAHHHLA     300
PsaA2         HEFILNTSLMAELYPSFAKGLVPFFTLQWGQYADFLTFKGGLNPVTGGLWLSDTAHHHLA     298

PsaA1         IAVLFIIAGHMYRTNWGIGHSLKEILENHKGPF--------TGDGHRGLFENMTTSWHAQ     352
PsaA2         LAVLFIVAGHFYRTNWGIGHSFKEMLDDAKSPNMLPFLNFIGPVGHEGLDKIFETSWHAN     358

PsaA1         LGTNLAMLGSLTIIVAHHMYAMPPYPYLATDYATQLSIFTHHMWIGAFCIVGGAAHATIF     412
PsaA2         LSIHLVQFGTASLLVAHHMYAMPPYPYLATDYATALSLFTHHVWIAGFCIVGGAAHAAIF     418

PsaA1         MVRDYDPATNMNNVLDRVLRHRDAIISHLNWVCMFLGFHSFGLYIHNDTMQALGRPQDMF     472
PsaA2         MVRDYDPAHHVNNILDRTLRHRDVIISHLAWVCQFLGFHSFAMYCHNDTMRAFGRPQDMF     478

PsaA1         SDTAIQLQPVFAQWVQNLHTLAP------------------------GSTAPNALEPVSY     508
PsaA2         SDTGIQLQPIFAQWIQHIHTAAVGAAQVAQPLGDVFGGVRGIELSGLGTTAPGIGAPVSY     538

PsaA1         AFGGGVLAVGGKVAMMPIALGTADFMIHHIHAFQIHVTVLILLKGFLFARNSRLIPDKAN     568
PsaA2         AWGGGMVAVGGKVAMMPIALGTADFLIHHIHAFTIHVTVLVLFKGVLFARGSRLVPDKAN     598

PsaA1         LGFRFPCDGPGRGGTCQVSGWDHVFLGLFWMFNTISIAVYHFSWKMQSDVWGTVDPDGTI     628
PsaA2         LGFRFPCDGPGRGGTCQVSAWDHVFLGLFWMYNSLSMVVFHFSWKMQSDVWGTVDSDGIV     658

PsaA1         NHITAGNWALSATTINGWLRDFQWAQAAQVIQSYGSALSAYGLLFLGAHFVWAFSLMFLF     688
PsaA2         THLTGGNFATSSITNNGWLRDFLWAQSAQVIQSYNSSLSAYGLMFLAGHFIFGFSLMFLF     718

PsaA1         SGRGYWQELIESIVWAHNKLKVAPTVQPRALSIIQGRAVGVAHYLLGAIVTIWAFFEARI     748
PsaA2         SGRGYWQELIESIVWAHNKLKVAPAIQPRALSIVHGRAVGVAHYLLGGIVTTWAFFLARM     778

PsaA1         LSVG  752
PsaA2         SAIG  782
```

B

```
PsaB1         MATKFPKFSQDLAQDPTTRRIWYGIATAHDFESHDGMTEENLYQKLFATHFGHLAIIFLW     60
PsaB2         MATKFPKFSQDLAQDPTTRRIWYAMATAHDFELHDGMTEENLYQKIFASHFGHLAIIFLW     60

PsaB1         ASSLLFHVAWQGNFEQWIKDPLHVRPIAHAIWDPQFGKAAVDAFTQGGASYPVNIAYSGV     120
PsaB2         ASGVLFHVAWQGNFEQWIKDPLNVRPIAHAIWDAQFGPPAIEAFTRAGATNPVDICYSGV     120

PsaB1         YHWWYTIGMRTNNDLYMGSVFLLLLASLFLFAGWLHLQPKFRPSLSWFKSAEPRLNHHLA     180
PsaB2         YHWWYTIGMRTNNELYVGAIFLLLLAALFLFAGWLHLQPRYRPTLGWFKSAEPRLNHHLA     180

PsaB1         GLFGVSSLAWTGHLVHVAIPESRGQHVGWSNFLTTPPHPDGLQPFFSGNWGAYAANPDTA     240
PsaB2         GLFGVSSLAWAAHLIHVAIPESRGQHVGWDNFLFTPPHPAGLGAFFTGNWSAYAQNPDTA     240

PsaB1         NHVFGTSQGAGTAILTFLGGFHPQTQSLWLTDMAHHHLAIAVLFIVAGHMYRTNFGIGHS     300
PsaB2         QHVFNSSQGAGTAILTFLGGFHPQTQSLWLTDMAHHHVAIAVLFIIAGHMYRTNWGIGHS     300

PsaB1         IKEMLNAKKFFGASTEGQFNLPHQGLYDTINNSLHFQLSLALAALGTITSLVAQHMYAMP     360
PsaB2         IKEMLNSKSFFGAKVEGPFNLPHQGLYDTINNSLHFQLSFALAALGVASSLTAQHMYSMP     360

PsaB1         PYAFIGQDFTTQAALYTHHQYIACALMLGAFAHAAIFWVRDYDPEQNKGNVLDRVLKHKE     420
PsaB2         PYAFIGQDFTTQAALYTHHQYIAGFLMVGAFSHAGIFWIRDYDPEQNKGNVLDRMLRHKE     420

PsaB1         AIISHLSWVSLFLGFHTLGLYVHNDVVVAFGTPEKQILIEPVFAQFIQGAHGKVLYGFDT     480
PsaB2         AIISHLSWVSLFLGFHTLGLYVHNDVEVAFGAAEKQVLIEPVFAQFIQAAHGKALYGFNT     480

PsaB1         LLSNPDSVASTA----GAAWLPNWLDAINNGTNSLFLTIGPGDFLVHHAFALAIHTTVLV     536
PsaB2         LLSNPDSIASTAWPNHANVWLPGWLDAVNNTTNSLFLTIGPGDFYVHHAIALGLHVTTLV     540

PsaB1         LVKGALDARGSKLMPDKKDFGYAFPCDGPGRGGTCDISAWDSFYLAAFWVLNTAGWVTFY     596
PsaB2         LVKGALDARGSKLMPDKKDFGYAFPCDGPGRGGTCDISAWDASYLAVFWMLNTLGWVTFY     600

PsaB1         WHWKHLGIWQGNVAQFNESSTYLMGWLRDYLWLYSAQLINGYNPYGMNNLSVWAWMFLLG     656
PsaB2         WHWKHLAIWEGNIAQFNESSTYLMGWFRDYLWLHSAQLINGYNPYGTNSLAIWSWMFLWG     660

PsaB1         HLIWATGFMFLISWRGYWQELIETLVWAHERTPLANLIRWKDKPVALSIVQARIVGLGHF     716
PsaB2         HLAWAVSFMFLITWRGYWQELIETLVWAHEKTPLS-FGYWRDKPVALSIVQARLVGLTHF     719

PsaB1         AAGYILTYAAFLIASTAGKFG  737
PsaB2         TVGYIATYGAFLIASTAGKFG  740
```

Fig. S5 Alignment of PsaA and PsaB homologs showing the location of shared tryptic peptides. Proteomic analysis most often employs upstream proteolysis with trypsin to generate peptide fragments for nano-flow liquid chromatography coupled on-line to mass spectrometry. Protein homologs with a significant degree of sequence identity may contain shared tryptic peptides. This situation is exemplified by isoforms of the photosystem I subunits (**A**) Chro_5026/1019 (PsaA1/A2) and (**B**) Chro_5027/1018 (PsaB1/B2), with shared peptides highlighted in colored rectangles. For quantification of differential expression by iBAQ, which utilizes the sum of all peptide ion intensities attributable to each protein, peptides with ion intensities contributed by more than one isoform may introduce inaccuracy. For relative quantification of photosystem subunit isoforms, we instead used the Top-N method in which the three most intense and unique tryptic peptide ion intensities mapping to a protein were summed to generate the abundance score.

```
6803      --------------MAESNQVVQAYNGDPFVGHLSTPISDSAFTRTFIGNLPAYRKGLSP 46
9212      MSNT------VDTIDTVDTDIIKSFKGDPCLGNLSTPINDSPLARAFINNLPAYRKGLTP 54
7203      MTNTANPSVESKLNYRLDTDPVQPYKGDPFNSNFSTAITDSPLARAFINNLPAYRKGLTP 60
7335      ---------MSASDAYISDDPIQPYQGNPQLGNLATPINSSNLAKAFINNLPAYRPGLTP 51

6803      ILRGLEVGMAHGYFLIGPWTLLGPLRDSEY-QYIGGLIGALALILVATAALSSYGLVTFQ 105
9212      FMRGLEIGMAHGYFLVGPEVVIGPLRESAHGANLSGLITAIYIAVSACLGISIFAITTFQ 114
7203      FMRGLEIGMAHGYFLVGPEVVVGPLRETAHGANLSGLITAIYIAVSACLGISIFAITTFQ 120
7335      FLRGLEIGMAHGYFLVGPEVVFGPLKEGSHGANLSGLITAIYITVSACLGISIFALATFQ 111

6803      GEQGS---------GDTLQTADGWSQFAAGFFVGGMGGAFVAYFLLENLSVVDGIFRGLF 156
9212      GNPKGSYSSYSTDSLRPLRSREEWSQLNGGIFLGAMGGAVFAYLLLENFDSLDAILRGAV 174
7203      GDPRGAYGSTSKDSLRPLRNRDEWYQLNGGIFLGAMGGAVFAYLLLENFDALDSILRGGV 180
7335      GDPRGTYNSHSRDRLRPLRKKEDWYQLSGGILMGSLGGAIFAYALLENFELLDSILRGAV 171

6803      N------ 157
9212      NVSYLPF 181
7203      NVN---- 183
7335      NVG---- 174
```

Fig. S6. Sequence alignments of PsaL from *Synechocystis* 6803 with FaRLiP encoded PsaL2 subunits from *C. fritschii* 9212, *C. thermalis* 7203 and *Synechococcus* 7335. The sequences for the FaRLiP encoded PsaL2 subunits that are incorporated into trimeric PSI complexes have been aligned with the PsaL subunit from *Synechocystis* 6803, which also forms trimeric complexes. The colored boxes show the four conserved amino acid residues that have been identified as important for trimer formation.

```
7120      --MAQAVDASKNLPSDPRNREVVFPAGRDPQWGNLETPVNASPLVKWFINNLPAYRPGLT 58
9212      --MAQAVDASKNSPSDPRNREVVFPAYRDPQVGNLETPINSSALVKWFIGNLPAYRPGIT 58
7203      --MAQAIDASKNRPGDPRNQEVVFPAGRDPQNSNLETPVNSSGLVKWFINNLPAYRPGIT 58
7335      ---------------MPASSNFIKPYEGDPQIGNLETPLNSSGLSKAFLENLPAYRTGLS 45
Chro_2988 --------------------DLIQPPLANPRSGDRFSSVEANDLTLNFLKYLPIYRPGIT 380


7120      PFRRGLEVGMAHGYFLFGPFAKLGP---LRDAANANLAGLLGAIGLVVLFTLALSLYANS 115
9212      TFRRGLEIGMAHGYWIFGPFAKLGP---LRNTVNANLAGLLATLGLIVILTGALSLYANS 115
7203      DMRRGLEVGMAHGYWVLGPFTKLGP---LRDTDVANIAGLISTLGMVAIMTATMALYSAS 115
7335      AQRRGLEVGMAHGYLLYGPFALLGP---LRDTDVLGITGLLSAIGLVLILTVCLSIYGGA 102
Chro_2988 PLSRGLEIGMAHGYWLVGPFTILGSLGSLNDSRASNLLGLLAAGSLIVILTIGFSIYGST 440


7120      NPPTALASV------TV-PNPPDAFQSKEGWNNFASAFLIGGIGGAVVAYFLTSNLALIQ 168
9212      NPPKPVNSV------TI-PNPPEAFQSNEGWNGFASAFLIGGIGGAVTAYFLTTNLALIQ 168
7203      NPPQPVATT------TTGGQVPSTFKSPESWNNYISGFLIGGVGGAVFAYFVLTNIAIIK 169
7335      DVSSEISRN------TLPYQPPEALSTDEGWSEFAGSFLIGGIGGAIFAYFLSANLPLLL 156
Chro_2988 SQEKSLVTVPRPNFAVTVPNVPDSLQAVDNWSQFSTGFFIGGIGGAIFAYLLLDNLNLFR 500


7120      GL--VG---- 172
9212      GL--FG---- 172
7203      NV--FGGLFS 177
7335      GS--IAGA-- 162
Chro_2988 AIPMIGS--- 507
```

Fig. S7. Sequence alignments of PsaL from Anabaena 7120 with non-FaRLiP encoded PsaL1 subunits from *C. fritschii* 9212, *C. thermalis* 7203, *Synechococcus* 7335 and the PsaL domain of the Chro_2988 protein from *C. thermalis* 7203. The sequences for the PsaL1 subunits preferentially expressed under WL growth conditions in addition to the PsaL domain of the Chro_2988 protein have been aligned to show the presence or absence of 2 multiproline motifs (highlighted in blue and purple), commonly found in PsaL subunits that do not form trimeric complexes.

```
9212-WL  ----------MAASFLPSIFVPLTGLVFPFVAMAFLFVYIEREDLV------------------------ 36
7203-WL  ---------MFSASFLPSILVPLTVLVFPSVAMALLFLYIEREDPSGI---------------------- 39
7335-WL  ----------MSASFLPTILVPTVGLVFPAIAMAALFLYIERGQATTGGESAPWGQVSEDSQ---TDVV- 56
9212-FRL MVDMTQLTGDYAASWLPWIMIPLIFYILPFPVFAILFLWIQKEDSEQIQETDSNLAKVGELEAPKP---- 66
7203-FRL MVDMTQLTGSYAASWLPWIMIPLIFYILPFPVFALIFIWIEKEAGTADEEV------------------- 51
7335-FRL MVDATQLEGAYAAAWLPWIMIPMITYILPFPIFAIAFLWIEREGGEGGLDIDVMGSNAMSNEAMGRDISS 70
```

Fig. S8. Sequence alignments of FaRLiP encoded and non-FaRLiP encoded PsaI subunits from *C. fritschii* 9212, *C. thermalis* 7203 and *Synechococcus* 7335. The sequences of both versions of the PsaI subunit from all three organisms investigated in this study were aligned to show the presence of a motif at the N-terminus of the PsaI2 proteins preferentially expressed under FRL growth conditions (orange) is absent in the PsaI1 subunits that are expressed under WL.

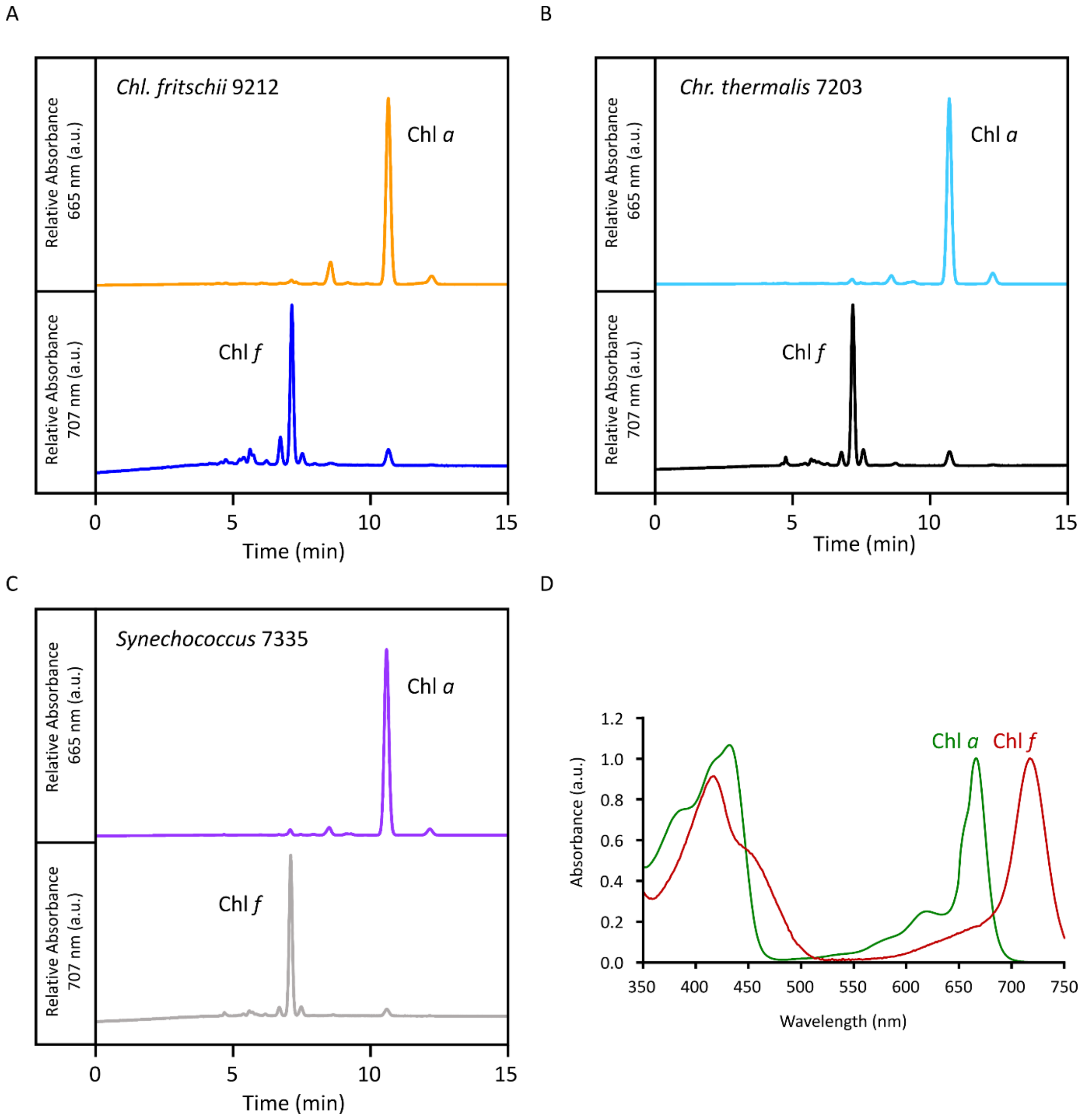

Fig. S9. Analysis of pigments from cyanobacteria grown under FRL conditions. Pigments extracted from FRL-acclimated membranes from (**A**) *C. fritschii* 9212, (**B**) *C. thermalis* 7203 and (**C**) *Synechococcus* 7335 were separated by HPLC. Absorbance was monitored at 665 nm (top panels) to show the presence of Chl *a* and 707 nm (bottom panels) to show the presence of Chl *f*. (D) The identity of the pigments were confirmed by their characteristic absorption spectra. HPLC traces (panels **A**-**C**) were normalised to Chl *a* and Chl *f* peaks and absorption spectra (panel **D**) were normalised to the Qy transition peak. In **A**-**C** 0 min corresponds to the start of isocratic elution in 100 % methanol.

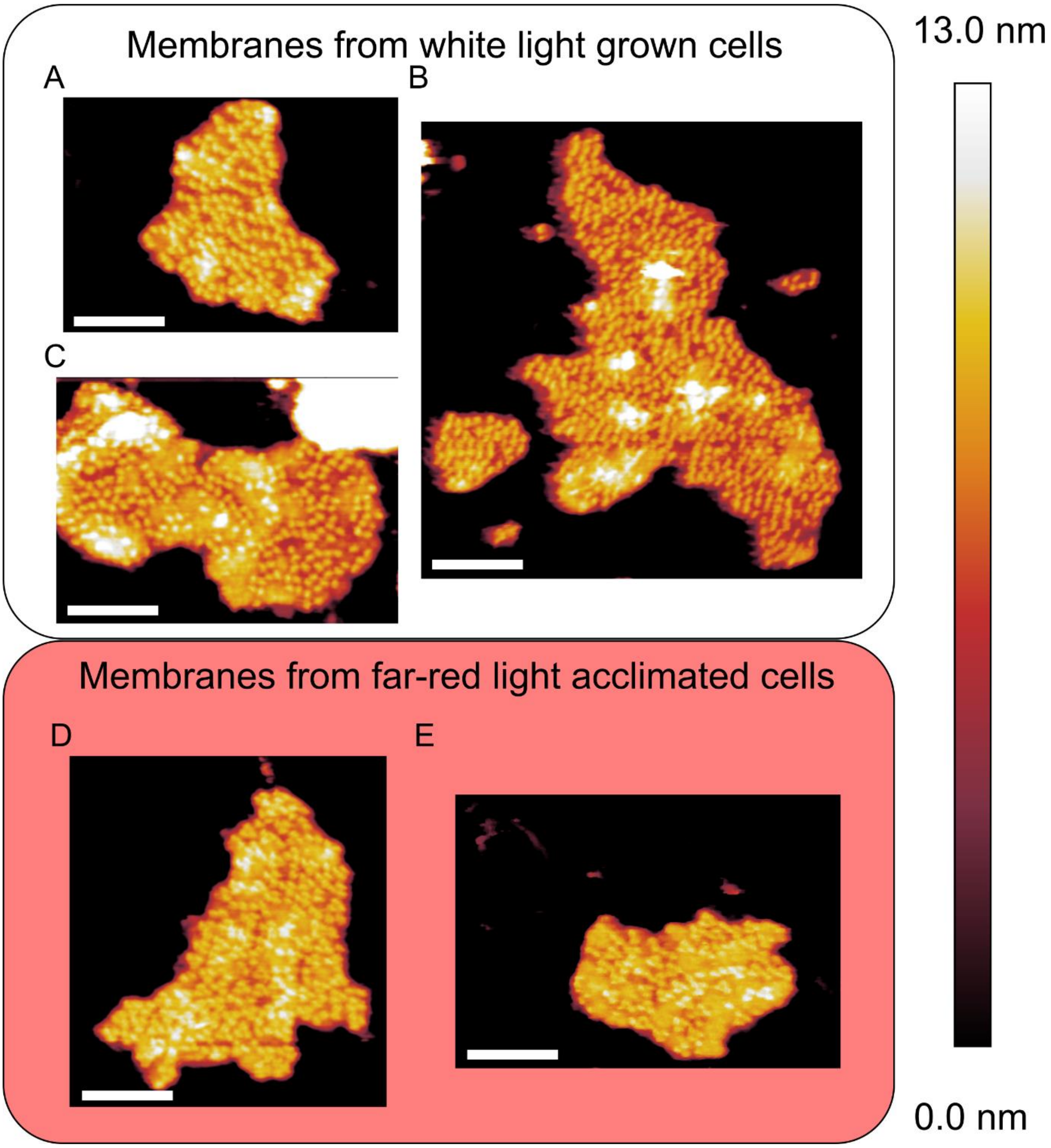

Fig. S10. Gallery of AFM images of thylakoid membranes from WL- and FRL-acclimated *C. thermalis* 7203. (**A**-**C**) AFM topographs of thylakoid membranes from *C. thermalis* 7203 grown under WL showing large areas of membrane containing PSI complexes in either a dimeric or tetrameric configuration. (**D**-**E**) Thylakoid membranes from FRL acclimated *C. thermalis* 7203 cells imaged by AFM showing PSI complexes in a trimeric configuration. Scale bars are 100 nm.

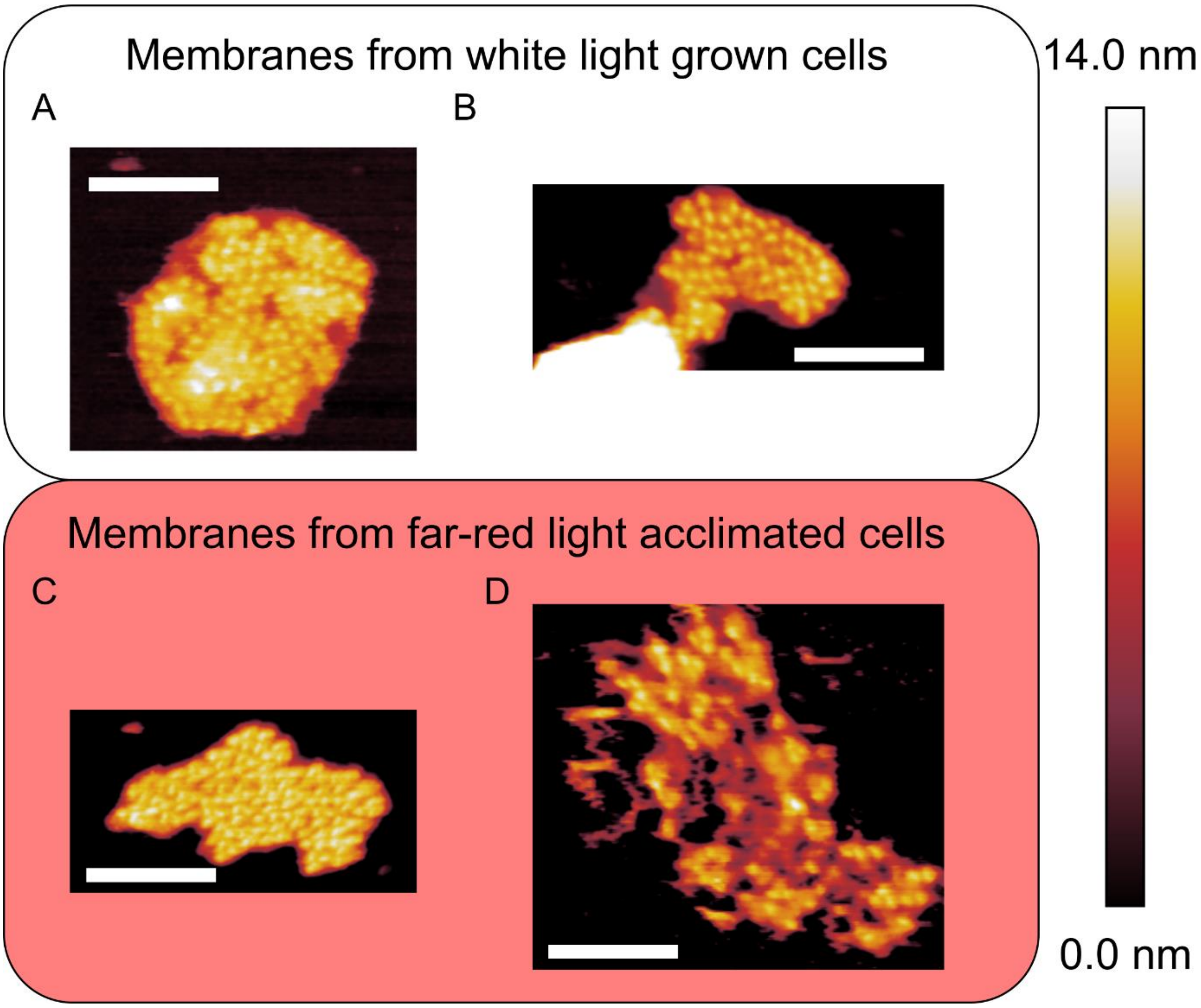

Fig. S11. Gallery of AFM images of thylakoid membranes from WL- and FRL-acclimated *C. fritschii* 9212. (**A** and **B**) Thylakoid membranes from *C. fritschii* 9212 grown under WL imaged by AFM showing PSI complexes in either a dimeric or tetrameric configuration. (**C**-**D**) AFM imaging of thylakoid membranes from FRL acclimated *C. fritschii* 9212 cells showing PSI complexes in a trimeric configuration. Scale bars are 100 nm.

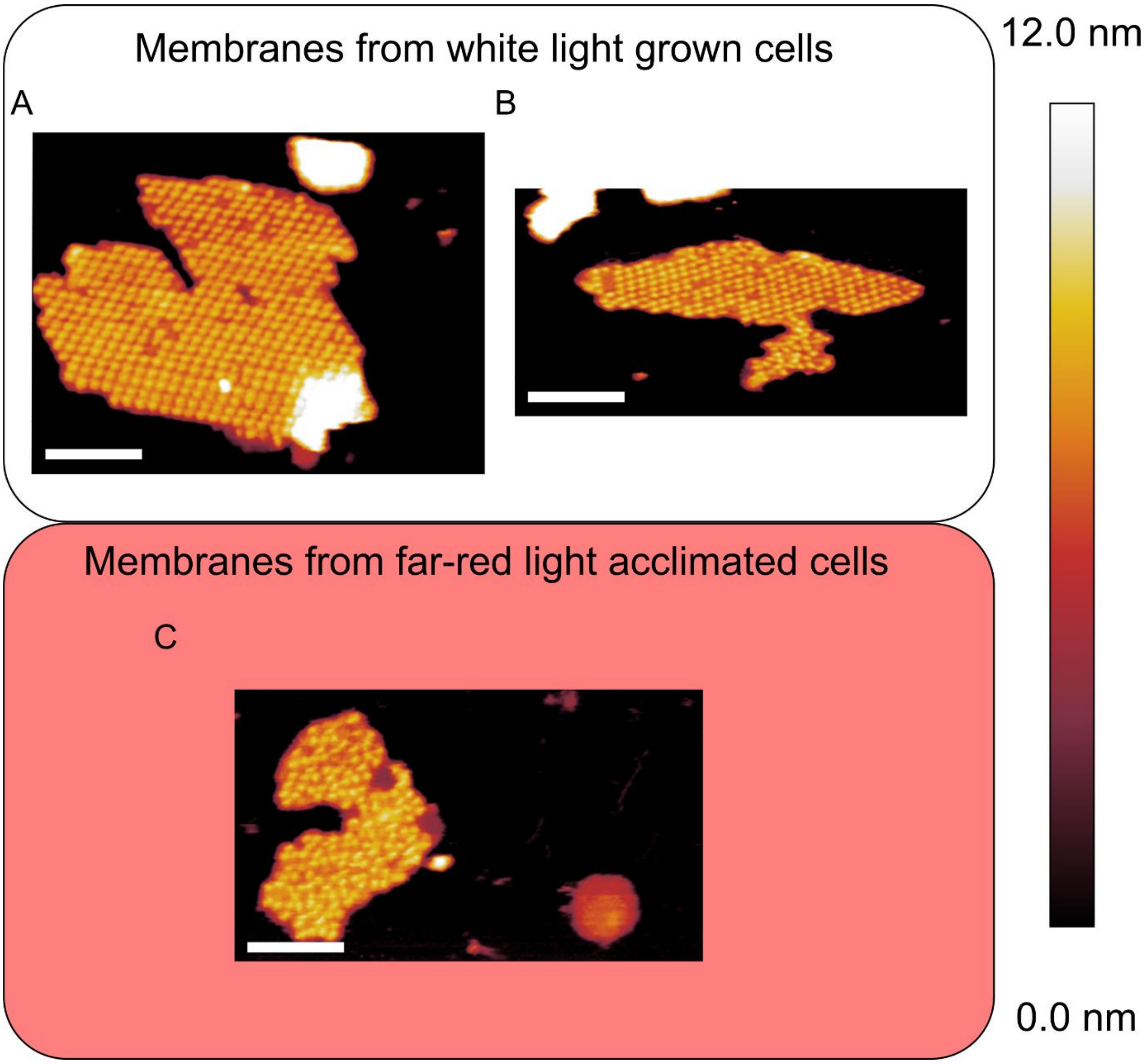

Fig. S12. Gallery of AFM images of thylakoid membranes from WL- and FRL-acclimated *Synechococcus* 7335. (**A** and **B**) *Synechococcus* 7335 thylakoid membranes isolated from cells grown under WL imaged by AFM showing PSI complexes in either a dimeric or tetrameric configuration forming pseudo-crystalline arrays. (**C**) Thylakoid membrane patch from FRL acclimated *Synechococcus* 7335 cells imaged by AFM showing PSI complexes in a trimeric configuration in a disordered array. Scale bars are 100 nm.

Data S1. (separate file)

This data set is from *Chroococcidiopsis thermalis* PCC7203 cells were acclimated under white and far-red light illumination. Proteins were extracted from thylakoid membranes and digested with endoproteinase Lys-C and trypsin. The resultant peptide fragments were analysed by nanoLC-MS/MS as three technical repeats (Rep 1, 2, 3) and the mass spectra subjected to database searching by MaxQuant as described in Materials and Methods.

Protein abundance scores were derived by the iBAQ (intensity-based absolute quantification) method, as implemented by MaxQuant. These values were normalized using Perseus to the intra-analysis sum of iBAQ scores to compensate for random variability arising from sample loading and MS full-scan/product ion scan data-dependent acquisition patterns. The normalized abundance scores were then transformed to log(2). Missing values resulting from the non-detection of proteins in some analyses were replaced by imputation of random values (shown in blue) derived from a normal distribution and weighted to simulate expected low abundance scores, as implemented in Perseus using the default parameters.

Statistical analysis in Perseus was by a modified t-test to provide the significance threshold at $p < 0.05$. The -log(10) p-values and differences (FRL - WL iBAQ score averages) provide the data-points for the volcano plots in Fig. 4A, C (main article).

Data S2. (separate file)

This Data Set is derived from the same MaxQuant output as Data S1 and is confined to the photosystem subunits that were identified and quantified in both white and far-red light thylakoid protein extracts.

Abundance scores were derived by the Top-N method using tryptic peptide ion intensity values calculated by MaxQuant. These values were normalized using Perseus to the intra-analysis sum of peptide ion intensities to compensate for the random variability arising as described above. Each protein abundance score was determined by summing the normalized intensity values for the 1, 2 or 3 most intense peptide ions, depending on the number of peptides identified.

After transformation of the summed intensity values to log(2), statistical analysis was carried out as described above with the -log(10) p-values and differences (FRL - WL Top-N score averages) used to provide the data-points for the volcano plot in Fig. 4B (main article).
